# Transcriptome changes associated with apple (*Malus domestica*) root defense response after *Fusarium proliferatum* f. sp. *malus domestica* infection

**DOI:** 10.1186/s12864-022-08721-3

**Published:** 2022-07-02

**Authors:** Yanan Duan, Shurui Ma, Xuesen Chen, Xiang Shen, Chengmiao Yin, Zhiquan Mao

**Affiliations:** College of Horticulture Science and Engineering, National Key Laboratory of Crop Biology, Shandong Agricultural University, Taian, 271018 Shangdong China

**Keywords:** Apple replant disease, *Fusarium proliferatum* f. sp. *malus domestica*, Root defense response, Secondary metabolism, Transcriptome analysis

## Abstract

**Background:**

Apple replant disease is a soilborne disease caused by *Fusarium proliferatum* f. sp. *malus domestica* strain MR5 (abbreviated hereafter as *Fpmd* MR5) in China. This pathogen causes root tissue rot and wilting leaves in apple seedlings, leading to plant death. A comparative transcriptome analysis was conducted using the Illumina Novaseq platform to identify the molecular defense mechanisms of the susceptible M.26 and the resistant M9T337 apple rootstocks to *Fpmd* MR5 infection.

**Results:**

Approximately 518.1 million high-quality reads were generated using RNA sequencing (RNA-seq). Comparative analysis between the mock-inoculated and *Fpmd* MR5 infected apple rootstocks revealed 28,196 significantly differentially expressed genes (DEGs), including 14,572 up-regulated and 13,624 down-regulated genes. Among them, the transcriptomes in the roots of the susceptible genotype M.26 were reflected by overrepresented DEGs. MapMan analysis indicated that a large number of DEGs were involved in the response of apple plants to *Fpmd* MR5 stress. The important functional groups identified via gene ontology (GO) and the Kyoto Encyclopedia of Genes and Genomes (KEGG) enrichment were responsible for fundamental biological regulation, secondary metabolism, plant-pathogen recognition, and plant hormone signal transduction (ethylene and jasmonate). Furthermore, the expression of 33 up-regulated candidate genes (12 related to WRKY DNA-binding proteins, one encoding endochitinase, two encoding beta-glucosidases, ten related to pathogenesis-related proteins, and eight encoding ethylene-responsive transcription factors) were validated by quantitative real-time PCR.

**Conclusion:**

RNA-seq profiling was performed for the first time to analyze response of apple root to *Fpmd* MR5 infection. We found that the production of antimicrobial compounds and antioxidants enhanced plant resistance to pathogens, and pathogenesis-related protein (PR10 homologs, chitinase, and beta-glucosidase) may play unique roles in the defense response. These results provide new insights into the mechanisms of the apple root response to *Fpmd* MR5 infection.

**Supplementary Information:**

The online version contains supplementary material available at 10.1186/s12864-022-08721-3.

## Background

Apple replant disease (ARD) is a major limitation to the establishment of economically viable orchards on replant sites. It is caused by the buildup and long-term survival of soilborne necrotrophic fungi (*Rhizoctonia* spp., *Fusarium* spp., and *Cylindrocarpon* spp.) and oomycetes (*Phytophthora* and *Pythium*) and can be aggravated by the lesion nematode *Pratylenchus penetrans* [1, 2]. *Fusarium* spp. is a major component of the ARD pathogen complex and has been identified in replanted orchard soils in China [3]. Plant infection is caused by *Fusarium* spp. penetrating the xylem vessels of the root system via wounds or cracks in the lateral roots. Colonization of the vascular tissues causes the infection to reach the stem or the entire plant, causing phloem blockage, internal stem discoloration, and plant wilt [4]. Infected plants are stunted, the tips and edges of the leaves turn yellow, and wilting, extensive chlorosis, and root tissue rot can occur, leading to plant death in severe cases [4].

The principal method to control ARD is pre-plan fumigation of orchard soils to eradicate ARD pathogens. However, this approach is limited due to environmental pollution, high cost, and short-lived effects [1, 2, 4]. Apple is a perennial woody plant, use of resistant rootstocks as a component for disease management might offer a durable and cost-effective benefit to tree performance than the standard practice of soil fumigation for control of ARD [2]. Although tolerance to individual components of the ARD pathogen complex has been detected in apple germplasm, such as M26, Malling-Merton (MM) 106, and MM111 rootstocks were more susceptible to the native populations of *Pythium* spp. resident to these orchard soils than Geneva series (G11, G16, G30) or Budagovsky 9 (Bud9) rootstock, and some rootstock genotypes are resistant (M.9) or susceptible (MM.106) to infection by *Phytophthora* [5, 6]. At present, the molecular defense response of apple root to ARD pathogens has not been carefully studied due to the complex etiology and the difficulty in phenotyping the disease resistance [2]. Therefore, understanding the molecular mechanisms of apple root resistance to ARD pathogens is necessary to implement a genetic-based breeding strategy for resistant apple rootstocks [7].

The root system is crucial for performing biological functions, such as absorbing water and nutrients, storing assimilates, and plant anchoring [8]. However, due to the lack of visual observation and standard phenotyping methods, investigating the molecular defense responses of roots interacting with soilborne necrotrophic pathogens is challenging [1, 3, 8, 9]. The current understanding of plant molecular defense responses is derived primarily from studies of foliar pathosystems [2, 10]. Due to advances in molecular techniques, RNA sequencing (RNAseq)-based transcriptome analyses have become a powerful tool for unraveling the global networks of transcriptional regulation and have been performed in numerous pathosystems [1–3, 10]. For example, Guo et al. [11] characterized the root transcriptome of *Gossypium barbadense* and provided gene resource (peroxidase (POD), GSH POD, aquaporin PIP, chitinase, L-ascorbate oxidase, and leucine rich-repeat (LRR) receptor genes) related to defense responses against *Verticillium dahliae*. Li et al. [12] found that * Fusarium oxysporum* f. sp. *cubense* infection induced the expression of many genes commonly responsive to infection by other pathogenic microorganisms, including PR genes (such as thaumatin-like genes), ethylene-responsive transcription factors (ERF), and genes involved in the synthesis of phytoalexins and phenolpropanoids (PAL) and cell wall strengthening (the gene encoding lignin-forming anionic peroxidase). Xiang et al. [3, 13] found that the synthesis of secondary metabolites and WRKY transcription factors (WRKY) had a unique role in the M9T337 apple rootstock resistance responses to *Fusarium solani*, and the *MdWRKY74* overexpression in apple callus significantly improved the resistance to *F. solani*. Shin et al. [14] used RNA-seq to identify transcriptomic changes associated with apple root defense responses to *P. ultimum* infection. It was found that these were principally associated with secondary metabolisms, cell wall fortification, and pathogenesis-related (PR) proteins, laccase, mandelonitrile lyase, and cyanogenic beta-glucosidase. All of these studies have shown that plant hormones (e.g., salicylic acid (SA), ethylene (ET), and jasmonic acid (JA)), oxidative burst, activation of secondary metabolism (e.g., phenylpropanoids), and many PR proteins are crucial for plant defense responses [1–4, 7, 11, 13]. Plant resistance to necrotrophic pathogens also involves the production of antimicrobial compounds and cell wall strengthening to limit pathogen progression and prevent cell death [10]. However, the specific defense activation mechanism in the roots of perennial tree crops like apple to soilborne pathogens has not been investigated by RNA-Seq [9].

In the early stage of this experiment, host-specific pathogenic fungi (*F. proliferatum* f. sp. *malus domestica* MR5) were isolated from the diseased roots of apple trees with ARD symptoms (weak growth or death) in replanted orchards in around the Bohai Gulf in China. These fungi were highly virulent to different apple seedlings [15]. The objectives of this research were to 1) analyze the transcriptional response of resistant and susceptible apple rootstock genotypes to *Fpmd* MR5 infection using RNA-Seq, 2) determine phenotype-related differentially expressed genes, and 3) study the molecular response of apple roots to *Fpmd* MR5 infection. The results will provide a deeper understanding of the mechanisms of apple root resistance to ARD-related pathogens.

## Materials and methods

### Culturing of *Fpmd* MR5

The *Fpmd* MR5 was isolated from the diseased root tissues of *Malus* × *robusta* (CarriŠre) Rehder apples grown in Shandong Province, China [16, 17]. *Fpmd* MR5 was cultured on potato dextrose agar (PDA) for 7 d at 28 °C. The cultured isolate was incubated in BVC medium (one vitamin b tablet, including VB1 3 mg, VB2 1.5 mg, VB6 0.2 mg, nicotinamide 10 mg, and calcium pantothenate 2 mg, vitamin C 0.1 g, KH_2_PO_4_ 1 g, KNO_3_ 1 g, sucrose 0.5 g, agar 20 g, and 1 L distilled water) for 7 d at 28 °C to obtain the conidia. The resulting spore suspension was diluted to approximately 1 × 10^6^ spores per mL with sterile distilled water prior to inoculation [12].

### Plant material and inoculation method

The replant tolerant rootstock Malling M9T337 (*Malus domestica* Borkh) [18] and the replant susceptible rootstock Malling 26 (M.26) [19] were purchased from Shandong Huinong Horticultural Technology Co., Ltd. (Shandong, China). The apple seedlings were propagated using a half-strength Murashige and Skoog (MS) medium containing 2% sucrose, 0.2 mg L^− 1^ of indole-3-acetic acid (IAA), 0.6 mg L^− 1^ of 6-benzyladenine, and 0.1 mg L^− 1^ of gibberellic acid (GA) (Solarbio, China). After the plants had rooted, they were grown in a mixture of soil and perlite (1:1). The plants were acclimatized for four weeks in a greenhouse and were grown in 12 h light/12 h dark conditions at 24 °C and 95% humidity [3].

The inoculation method described by Shin et al. [14] was used. The seedlings were inoculated with *Fpmd* MR5 by dipping the root system into the inoculum for 2 h and planting them in the aseptic soil/perlite mixture. The plants were watered thoroughly. The control plants were mock-inoculated with sterile distilled water, transplanted, and maintained under the same conditions as the pathogen-infected plants.

### Determination of the plant tissue collection time

**Fig. 1:**
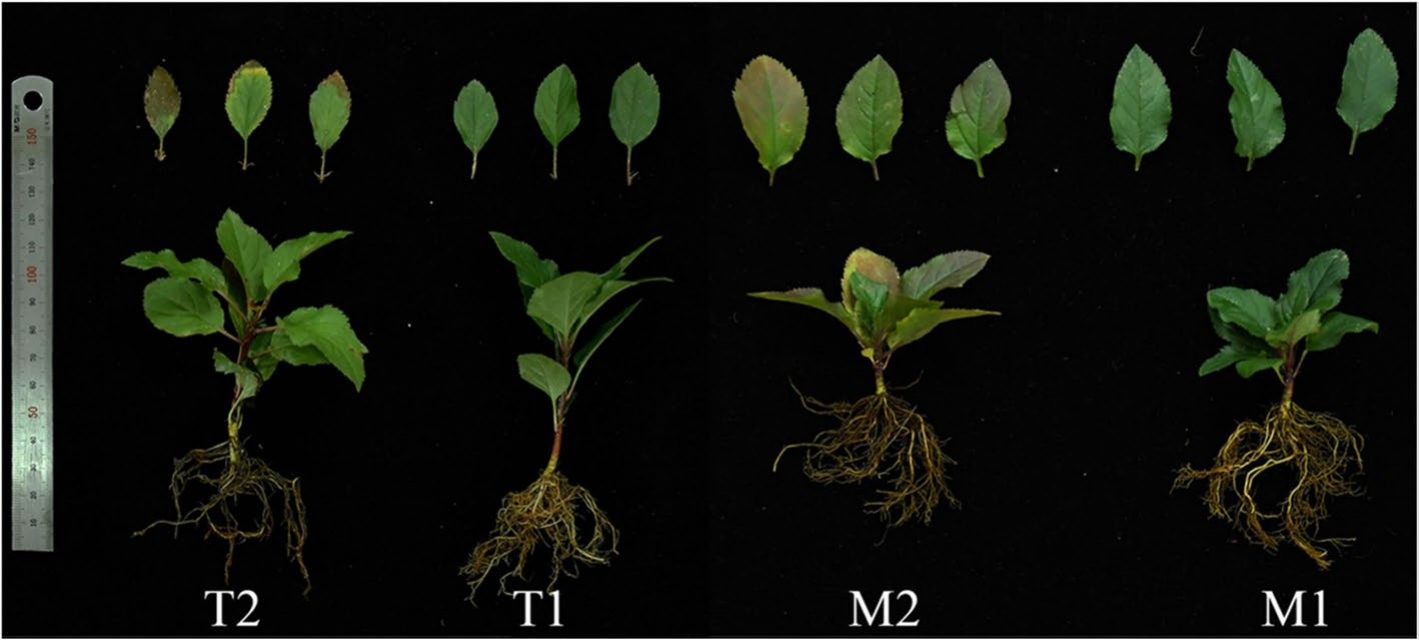
Response of M9T337 (T) and M.26 (M) plants with mock inoculated (T1 and M1) and *Fpmd* MR5 infected (T2 and M2)

We determined the appropriate time to collect the plant material after inoculation with *Fpmd* MR5 to obtain the apple root defense genes. The number of DEGs with double-digit fold changes were identified from *P. ultimum* inoculated apple root tissues at 48, 72, and 96 h because these periods have shown to be suitable to obtain defense-related genes [14]. We performed root tissue cultivation on different days post-inoculation (dpi) (1, 2, 3, 4, 5, 6, and 7 dpi) to observe the apple seedling response to *Fpmd* MR5. We used the fungal recovery assay described by Fradin et al. [20] with minor modifications and obtained root tissues from three inoculated plants at different dpis. The surface was sterilized with 70% ethanol for 15 min, followed by 15 min in 10% hypochlorite, rinsing three times with sterile water, and slicing. Ten slices of each plant were transferred onto PDA supplemented with streptomycin (50 mg L^− 1^) and incubated at 28 °C. *Fpmd* MR5 mycelia were most frequently observed on the cultured root tissues collected from M9T337 on 5, 6, and 7 dpi, while no mycelium was observed on 1, 2, 3, and 4 dpi. *Fpmd* MR5 mycelia were most frequently observed on the cultured root tissues collected from M.26 on 3, 4, 5, and 7 dpi, while no mycelium was observed on 1 and 2 dpi. As shown in Fig. 1, the M.26 plants began to show disease symptoms on the 3rd day, and the leaves gradually turned yellow, exhibiting chlorosis. The M9T337 plants began to develop disease symptoms on the 5th day, and the leaves turned yellowish-brown at the edge. These results indicated that the M9T337 roots and the M.26 roots could be collected after 4 d and 2 d, respectively, for obtaining apple root defense-related genes. The roots of the pathogen-infected and control seedlings were removed from the soil, washed with water, and flash-frozen in liquid nitrogen. The root tissue of twenty seedlings was collected, and the resulting data were pooled for each collection time per treatment. The experiment was repeated twice, and the pooled root tissues from the same collection time after inoculation were used for RNA isolation and RNA-seq analysis. The frozen root tissues were stored at − 80 °C. The subsequent transcriptome data were analyzed by two-way comparisons, i.e., comparisons within the tissue series (mock-inoculated and *Fpmd* MR5 infected) and between the two tissue series for the same treatment (Fig. S1, arrows).

### RNA-Seq library preparation and sequencing

Total RNA was extracted from each root sample using Trizol Reagent (Invitrogen, Life Technologies, Carlsbad, CA, USA) following the manufacturer’s protocol. The quantity and quality of the isolated root RNA were examined using a 2100 bioanalyzer (Agilent Technologies, Inc., Santa Clara, CA) [21]. A total of 3 μg RNA per sample was used as input material for the RNA sample preparations. Sequencing libraries were generated using an NEBNext^®^ Ultra™ RNA Library Prep Kit for Illumina^®^ (NEB, USA), following the manufacturer’s recommendations, and index codes were added to attribute sequences to each sample. The library fragments were purified with the AMPure XP system (Beckman Coulter, Beverly, USA) to select cDNA fragments with a length of 370-420 bp. Then polymerase chain reaction (PCR) was performed with Phusion high-fidelity DNA polymerase, universal PCR primers, and an index (X) primer. PCR products were purified (AMPure XP system), and library quality was assessed on the Agilent Bioanalyzer 2100 system [21]. Clustering of the index-coded samples was performed on a cBot Cluster Generation System using a TruSeq PE Cluster Kit v3-cBot-HS (Illumia) according to the manufacturer’s instructions [22]. After cluster generation, RNA library preparations were sequenced on an Illumina HiSeq PE150 platform, and 150 bp paired-end reads were generated at Novogene Bioinformatics Technology Co., Ltd., Beijing, China (www.novogene.cn).

### Sequence data and differentially expressed gene analysis

The clean reads were retrieved after trimming the adapter sequences and removing low-quality reads (containing > 50% bases with a Phred quality score < 20) and reads with unknown nucleotides (more than 1% ambiguous residues N) using the FastQC tool (http://www.bioinformatics.babraham.ac.uk/projects/fastqc/). A GC content distribution check was performed. All the downstream analyses were based on high-quality clean data [4]. The apple reference genome *Malus domestica* GDDH13 Whole Genome v1.1 was downloaded from the Genome Databases for Rosaceae (GDR, http://www.rosaceae.org) [1]. An index of the reference genome was established, and the paired-end clean reads were aligned to the reference genome using Hisat2 v2.0.5. The mapped reads of each sample were assembled by StringTie (v1.3.3b) using a reference-based approach. FeatureCounts v1.5.0-p3 was used to count the reads mapped to each gene [23]. The expected number of fragments per kilobase of transcripts per million mapped reads (FPKM) of each gene was calculated based on the length and mapped read count for that gene [3]. We used the transcription factor (TF) database (PlantTFDB) and protein domain database (Pfam/SUPERFAMILY) to predict the family based on the gene TF [24]. Differential expression analysis of the two groups was performed using the DESeq2 R package (v.1.20.0). The resulting *P* values were adjusted using Benjamini and Hochberg’s approach for controlling the false discovery rate [21].

### Functional annotation of candidate genes

Blast2go software was used with an E-value ≤1e-5, to annotate the DEGs’ major gene ontology (GO) categories, including molecular functions, biological processes, and cellular components. In addition, we performed KEGG (Kyoto Encyclopedia of Genes and Genomes) enrichment analysis on DEGs using the online KEGG Automatic Annotation Server (www.kegg.jp/kegg/kegg1.html) [7, 25].

### Quantitative RT-PCR analysis

The primers were designed using the National Center for Biotechnology Information (NCBI) primer blast, an online primer design tool (https://www.ncbi.nlm.nih.gov/tools/primer-blast/) [26] (Table S1). The frozen tissue samples were ground to a fine powder in liquid nitrogen, and the total RNA was extracted using the FastPure^®^ Plant Total RNA Isolation Kit (Vazyme, Nanjing, China). We used the HiScript^®^ III RT SuperMix for qPCR (+gDNA wiper) (Vazyme, Nanjing, China) to remove the genomic DNA from the total RNA (1000 ng RNAs of each sample) and synthesized cDNA. The PCR mixture contained 10.0 μL 2 × Taq Pro Universal SYBR qPCR Master Mix (Vazyme, Nanjing, China), 7.2 μL ddH_2_O, 0.4 μL of each gene-specific primer (10 μM), and 2 μL cDNA template. The qRT-PCR assays were performed using the CFX96 Touch™ RT-PCR Detection System (Bio-RAD, USA) with the following program: 95 °C for 30 s; 40 cycles of 95 °C for 10 s, 60 °C for 30 s, and 72 °C for 30 s. A commonly used reference gene, glyceraldehyde-3-phosphate dehydrogenase (GAPDH), was used to normalize the expression levels of the target genes [27]. The relative expression levels of the target genes were calculated with the 2^−∆∆CT^ method [3, 14]. Three biological replicates and three technical replicates were used for each of the selected genes.

### Data analysis

Principal component analysis (PCA) and KEGG pathway analysis were performed, and a correlation heatmap was created in R software (www.r-project.org). TBtools software was used for cluster analysis. The similarities and differences between the treatments were illustrated using a color gradient where the color intensity is directly proportional to the numerical value. The DEGs were annotated using the MapMan software.

## Results

### Sequencing data and transcriptome mapping

RNA-seq of the 12 root samples produced 518.1 million raw reads or an average of 43.2 million 150 bp paired-end reads per sample with Q30 bases (those with a base quality greater than 30) greater than 91% and Q20 bases (those with a base quality greater than 20) greater than 96%. An average ‘G + C’ content of above 46% was obtained (Table S3). After quality control, the reads were mapped to the apple genome sequence (*Malus domestica* GDDH13 Whole Genome v1.1). Overall, 84.62% of the reads were mapped to the draft apple genome sequences (Table S2). Among the selected reads, 79.93% of the control sample and 89.24% of the treatment sample were aligned to the apple reference genome, providing either unique matches or multiple matches to the genomic locations.

### Identification of DEGs responding to *Fpmd* MR5 infection

**Fig. 2:**
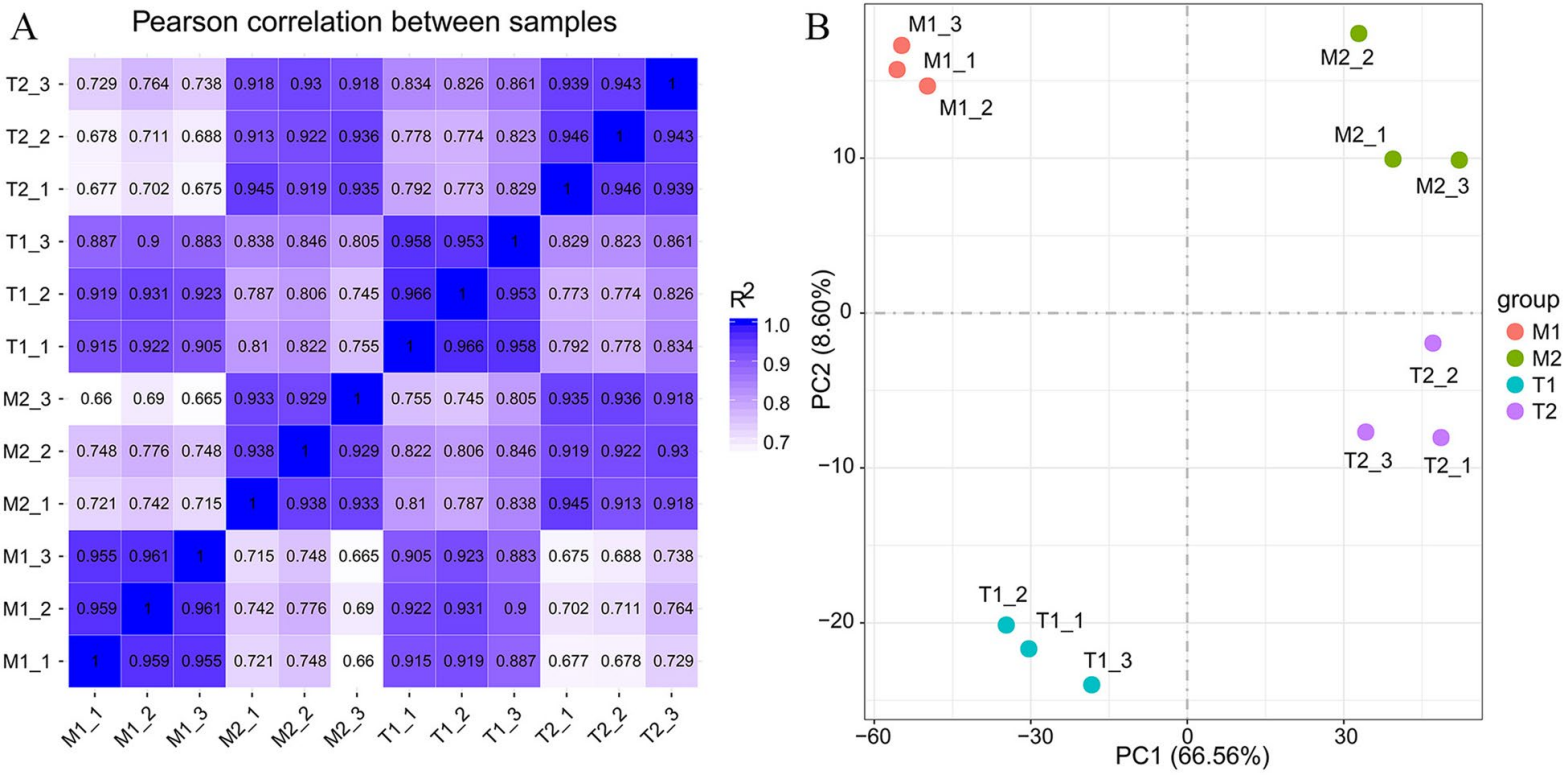
**A** The inter-sample correlation heatmap is based on pearson correlation. **B** Principal Component Analysis of different samples. M1 and T1: M9T337 (T1) and M.26 (M1) plants with mock inoculated. M2 and T2: M9T337 (T2) and M.26 (M2) plants with *Fpmd* MR5 infected. Three biological replicates per treatment

After calculating the expression value (FPKM) of the genes in each sample, we plotted the gene expression levels using box plots, violin plots, and probability density distribution diagrams. We found differences in gene expression levels between the treatment group and the control group and similarities between the treatment groups (Fig. S2). The PCA results and correlation heat maps showed that the biological repeatability of the samples within the group was high (R^2^ > 0.88), and differences occurred between the samples (M1 and M2, T1 and T2) and between the groups. The results indicate that the sample selection is reasonable and can be used for the subsequent differential gene analysis (Fig. 2). Among 46,558 discovered transcripts from the root samples, 7.34% were identified as DEGs (using a cutoff value of |log2 (fold change)| > 1 & padj <= 0.05). Two-way data analysis was performed for the cross-examination of the transcriptomic changes associated with *Fpmd* MR5 infection in apple root tissue (Fig. S3A). Significantly different expression levels were observed between the mock-inoculated and the *Fpmd* MR5 inoculated samples in 28,196 genes. Among those genes, 14,572 genes were up-regulated, and 13,624 genes were down-regulated in the *Fpmd* MR5 inoculation samples. It is worth noting that the number of DEGs in the replant susceptible rootstock M.26 treatment group was significantly higher than those in the replant tolerant rootstock M9T337. Physiological perturbation by the inoculation, the rootstock variety, and seedling repotting may have caused a larger number of identified DEGs in the mock-inoculated groups (T1 and M1). Therefore, we excluded the DEGs identified in the mock-inoculated groups and conducted a follow-up study using only the DEGs identified in the *Fpmd* MR5 infected group (Fig. S3B). The heatmap showed that the gene expression patterns were similar for T2, M2, and T1, M1 (Fig. S3C). K-means clustering was performed to obtain 4 groups of DEGs. The genes in the same cluster had similar trends of expression levels for different treatment conditions (Fig. S3D).

### Gene ontology analysis: functional categorization of identified differentially expressed genes

**Fig. 3:**
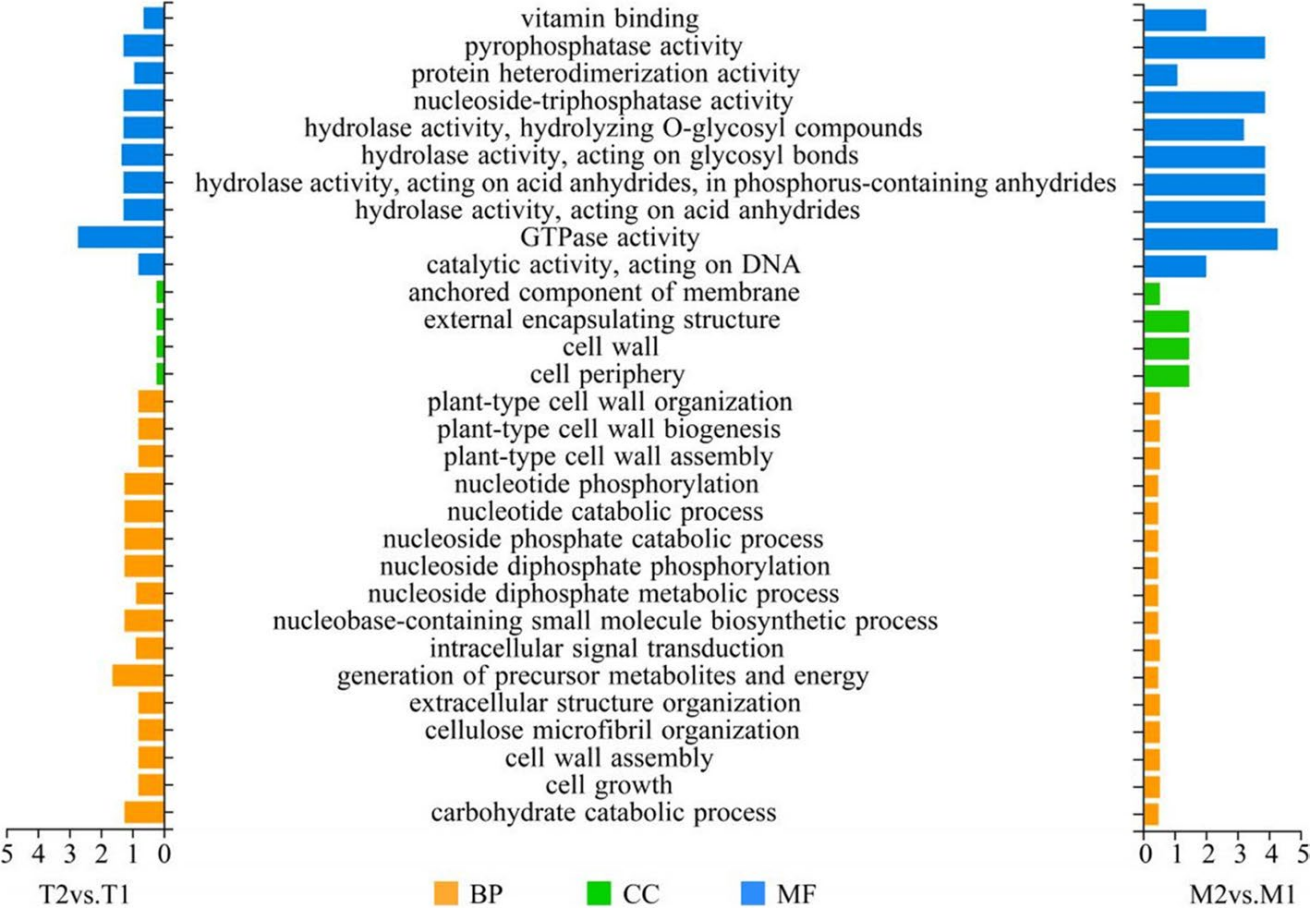
Gene Ontology (GO) categories of DEGs between mock inoculated and *Fpmd* MR5 infected (M2 and M1,T2 and T1). BP: biological process. CC: cellular component. MF: Molecular Function

We used clusterProfile software to perform GO functional enrichment analysis (Fig. 3). The cumulative numbers of the DEGs in the M2vs.M1 and T2vs.T1 datasets were grouped into three categories: biological processes, molecular functions, and cellular components. The most enriched sub-categories of the biological process were carbohydrate catabolic process, nucleotide catabolic process, intracellular signal transduction, and cell wall organization or biogenesis. Most of the mapped DEGs in the cellular component category were classified into a few sub-categories, including cell periphery, cell wall, anchored component of membrane, and external encapsulating structure. More than 30% of all DEGs were assigned to the cellular component of the membrane-related categories. This result highlights the increased cross-membrane activities in apple root tissues in response to *Fpmd* MR5 infection. The most enriched sub-categories in the molecular function category were DEGs with the annotated function of GTPase activity, hydrolase activity, pyrophosphatase activity, and nucleoside-triphosphatase activity. It is noteworthy that many of the up-regulated DEGs in the T2vs.T1 dataset were classified as biological processes, whereas many of the up-regulated DEGs in the M2vs.M1 dataset were classified as cellular components in the membrane-related categories (Fig. S4). Many down-regulated DEGs were assigned to the molecular function category, including nucleoside-triphosphatase activity, pyrophosphatase activity, GTPase activity, and hydrolase activity. Overall, we observed a considerable shift in the cellular functions related to metabolic pathways, energy production, transmembrane transport, and cell wall structure and function in the apple roots in response to the *Fpmd* MR5 infection.

### KEGG pathway analysis

**Fig. 4:**
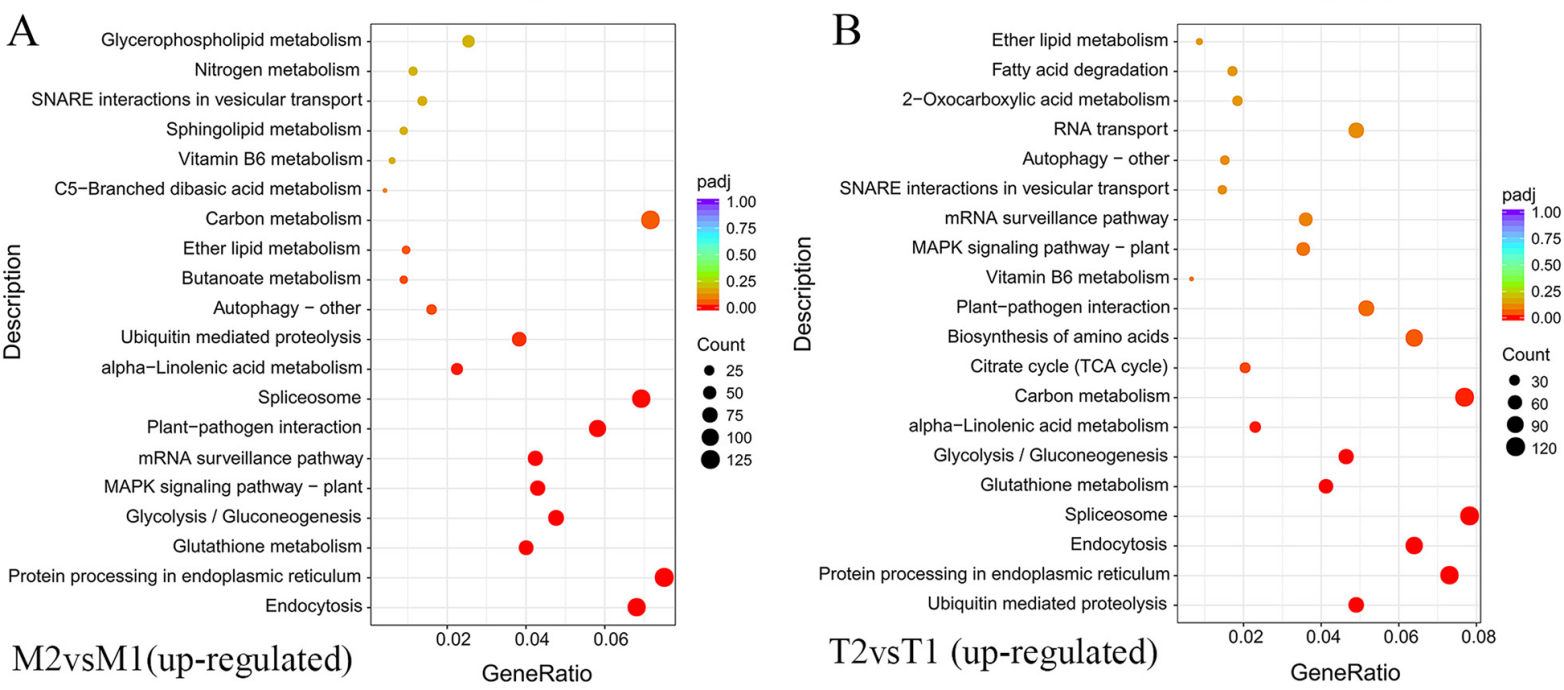
**A**-**B** KEGG terms enriched in up-regulated pathways between the mock inoculated and *Fpmd* MR5 infected. The abscissa is the enrichment factor (Ratio of differential genes enriched to this pathway to background genes of the pathway). The ordinate is the path description, the bubble size indicates the number of different genes, and the bubble color indicates the significance level of the *p*-value

KEGG pathway enrichment analysis was performed to categorize the biological functions of the DEGs (Table S4). The most noticeable change was an increased number of DEGs mapped to multiple pathways related to secondary metabolisms, such as starch and sucrose metabolism, carbon metabolism, and glycolysis/gluconeogenesis. Many up-regulated DEGs were assigned to endocytosis, protein processing in the endoplasmic reticulum, GSH metabolism, glycolysis/gluconeogenesis, mitogen-activated protein kinase (MAPK) signaling pathway, mRNA surveillance pathway, carbon metabolism, and plant-pathogen interaction (Fig. 4). The highest number of DEGs in the M2vs.T2 dataset were mapped to the nitrogen metabolism and phenylpropanoid biosynthesis (Table S4). These results suggest that the nitrogen metabolism, phenylpropanoid biosynthesis, carbon metabolism, plant hormone signal transduction, protein processing in the endoplasmic reticulum, and plant–pathogen interaction pathways were most affected in the plants infected with *Fpmd* MR5. These pathways are similar to the major pathways involved in plant and pathogen interactions in previous reports [3, 7, 14].

### MapMan analysis

**Fig. 5:**
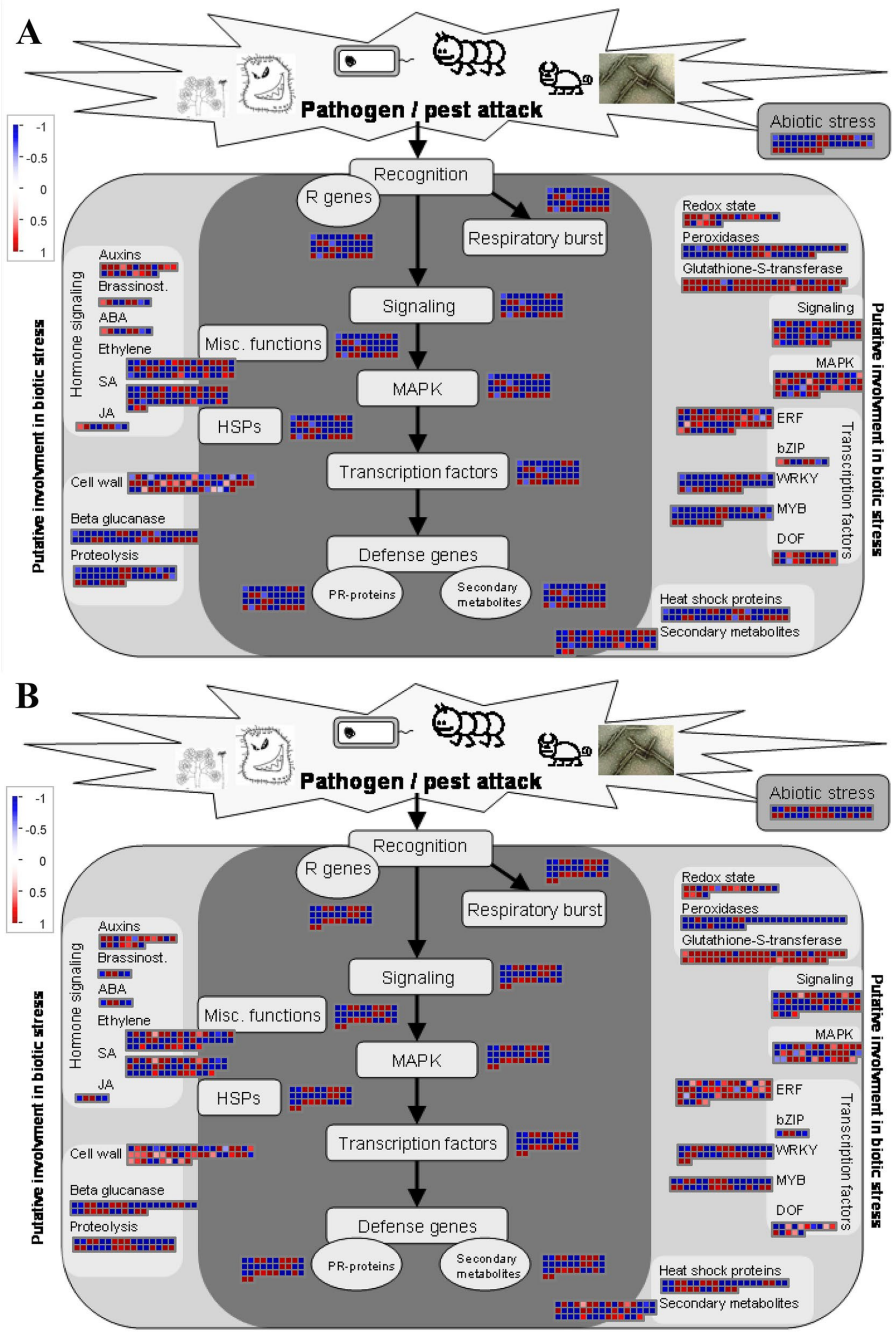
MapMan graphs of biotic stress in M2vs.M1 (**A**) and T2vs.T1 (**B**) datasets. The scale bar represents the log2FoldChange of the DEGs. Red and blue indicate upregulated and down regulated genes, respectively

The MapMan analysis indicated that the defense response of *Fpmd* MR5 to apple root infection occurred primarily through the activation of phytohormone biosynthesis (auxin, brassinolide, ABA, ethylene, JA, and SA), transcription factors (WRKY, MYB, and ERF), and the MAPK signaling pathway, leading to the activation of a large number of defense genes related to PR proteins and antimicrobial secondary metabolism, thereby improving plant resistance to *Fpmd* MR5 infection. The R protein genes (MD16G1174700, MD12G1122800, and MD07G1002800) in the ETI pathway and cell wall-strengthening genes (MD05G1252000 and MD13G1126900) were also significantly up-regulated (Fig. 5).

Further analysis of the regulatory pathways revealed that a large number of DEGs were annotated as receptor kinases. Genes related to the calcium regulation and encoding proteins were also upregulated (Fig. S5). The biosynthesis of terpenoids, lignins, flavonoids, phenylpropanoids, and glucosinolates were the primary secondary metabolic pathways (Fig. S6). Interestingly, a large number of DEGs were up-regulated in the shikimate and antioxidant production (glutathione, flavonols, anthocyanidins) pathways (Fig. S7). We also observed significant up-regulation of a large number of DEGs involved in the synthesis of beta-glucosidase, peroxidase, UDP-glycosyltransferase, beta 1,3 glucan hydrolases, and nitrilases, which are crucial for improving plant resistance (Fig. S8).

### Transcription factors encoding DEGs induced by *Fpmd* MR5 infection

**Fig. 6:**
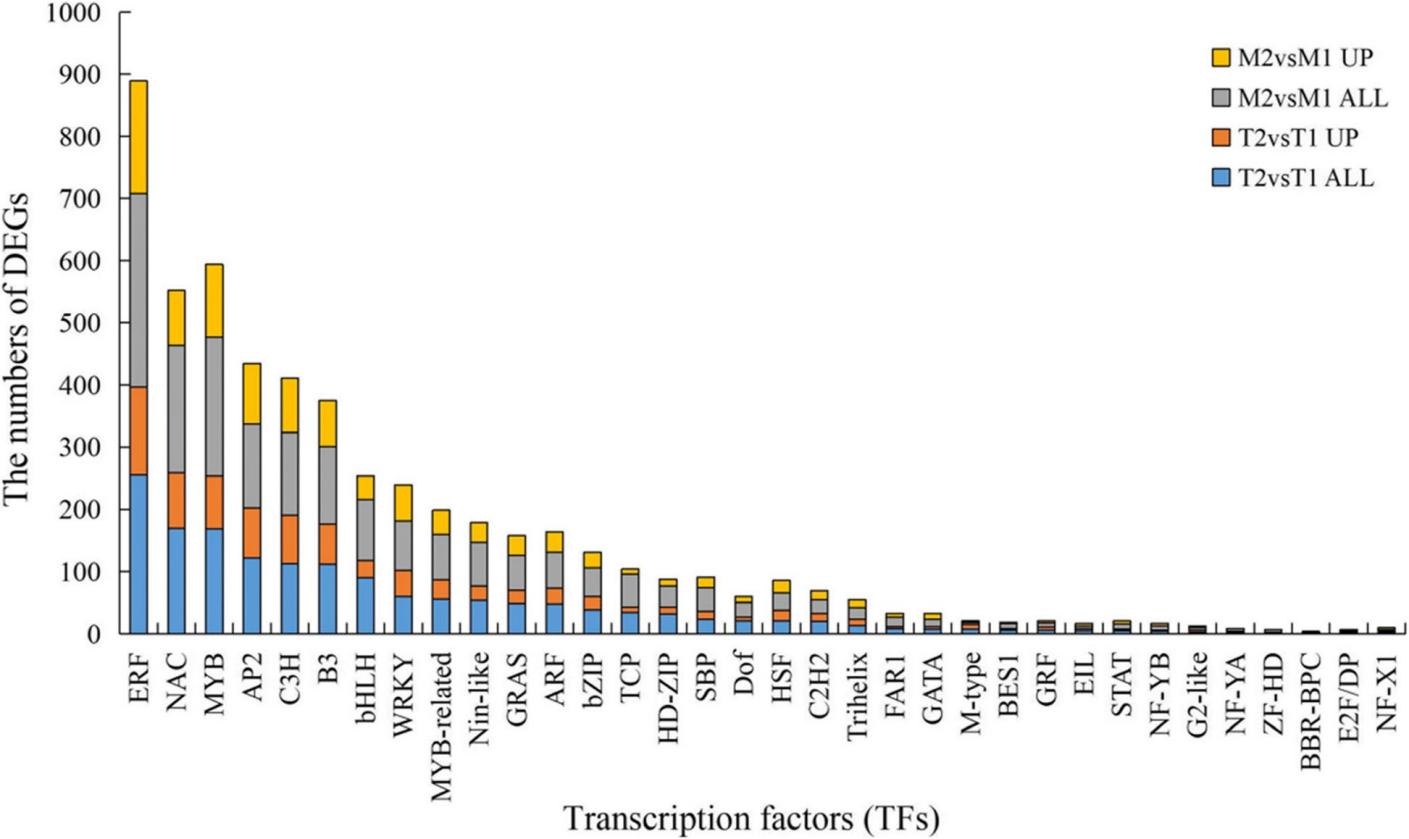
Identified DEGs which encode the transcription factors (TFs). X axis represents TFs, Y axis represents numbers of DEGs and up-regulated DEGs that specifically responded to *Fpmd* MR5 infection. Samples were represented by different colors

Many putative TFs encoding DEGs were identified (Fig. 6). The most enriched TF families included ethylene response factors (ERFs), NAC, MYB, AP2, C3H, B3, WRKY, and bHLH. More than 60 DEGs were identified, and they were similar in almost all treatments. Other TF encoding gene families were also considerably enriched, such as MYB-related, Nin-like, GRAS, ARF, bZIP, HD-ZIP, TCP, SBP, Dof, HSF, C2H2, and Trihelix. This result agreed with the findings of Xiang et al. [3], who found that many MYBs and ERFs showed higher transcription levels in the roots of *F. solani* infected M9T337 rootstock. Several of these TF gene families, such as WRKY, NAC, bHLH, and ERF, have also been identified as plant defense responses in multiple foliar pathosystems [28].

### Identification of DEGs involved in defense responses

DEGs encoding enzyme functioning in the carbon metabolism pathway were exclusively identified from *Fpmd* MR5 infected tissue and consistently showed up-regulation. Some showed double-digit fold increases in the expression level. These enzymes included *Malate dehydrogenase (MDH)* (MD10G1115300 and MD02G1243100), *Phosphoribulokinase* (MD06G1023500), *Phosphoenolpyruvate carboxykinase* (MD15G1405400), *L-3-cyanoalanine synthase* (MD01G1065600), *Serine acetyltransferase* (MD11G1202100), *Fructose-bisphosphate aldolase* (MD12G1018700 and MD12G1018800), *GAPDH* (MD17G1017300), *Alanine aminotransferase* (MD17G1154300), and *ATP-dependent 6-phosphofructokinase* (MD08G1109700 and MD15G1089400) (Table 1)*.*

**Table 1:** Carbon metabolism (mdm01200)

GeneName	KEGG	Function	log2FoldChange	
			T2vsT1	M2vsM1
MD17G1154300	mdm:103443626	Alanine aminotransferase	2.95	4.50
MD11G1202100	mdm:114819605	Serine acetyltransferase	3.9	5.00
MD06G1023500	mdm:103436834	Phosphoribulokinase	3.26	3.91
MD12G1018700	mdm:103449400	Fructose-bisphosphate aldolase	2.46	3.15
MD12G1018800	mdm:103449399	Fructose-bisphosphate aldolase	2.05	2.25
MD01G1065600	mdm:103405317	L-3-cyanoalanine synthase	4.01	3.87
MD15G1089400	mdm:103400183	ATP-dependent 6-phosphofructokinase	2.53	2.36
MD08G1109700	mdm:114826515	ATP-dependent 6-phosphofructokinase	2.63	2.45
MD10G1115300	mdm:103436249	NAD-malate dehydrogenase	6.92	8.17
MD17G1017300	mdm:103404229	Glyceraldehyde-3-phosphate dehydrogenase	3.14	3.83
MD15G1405400	mdm:103417932	Phosphoenolpyruvate carboxykinase	2.51	2.85
MD02G1243100	mdm:103408309	NAD-malate dehydrogenase	4.84	2.95

Multiple DEGs with the annotated function phenylpropanoid biosynthesis pathways were also mostly up-regulated (Table 2). Examples include *POD* (MD15G1321200, MD05G1306500, and MD05G1069000), *coniferyl-aldehyde dehydrogenase* (MD15G1187300), *4-coumarate--CoA ligase* (MD07G1309000), *scopoletin glucosyltransferase* (MD07G1007400 and MD00G1046200), *trans-cinnamate 4-monooxygenase* (MD03G1050900 and MD00G1221400), *Beta-glucosidase* (MD02G1242200), and *Caffeoyl-CoA O-methyltransferase* (MD02G1073400). These pathways are similar to those involved in the apple root response to *P. ultimum* infection [12]. It is worth noting that MD17G1265200 (*POD*) and MD01G1229000 (*Caffeic acid 3-O-methyltransferase*) only showed high expression levels in the replant tolerant rootstocks M9T337. MD03G1110800 and MD17G1224900 (*Shikimate O-hydroxycinnamoyltransferase*) showed high expression levels in the replant susceptible rootstock M.26. It was interesting that members of the ABC transporter family in the *ABC transporters* pathway showed high expression levels in the M2vs.M1 dataset (Table S5). They may play an important role in delivering antimicrobial secondary metabolites to the extracellular location of host-pathogen interactions [29].

**Table 2:** Phenylpropanoid biosynthesis (mdm00940)

GeneName	KEGG	Function	log2FoldChange	
			T2vsT1	M2vsM1
MD17G1224900	K13065	Shikimate O-hydroxycinnamoyltransferase ([EC:2.3.1.133])	1.89	3.97
MD15G1321200	K00430	Peroxidase ([EC:1.11.1.7])	1.42	3.71
MD15G1187300	K12355	coniferyl-aldehyde dehydrogenase [EC:1.2.1.68]	1.47	1.53
MD07G1309000	K01904	4-coumarate--CoA ligase ([EC:6.2.1.12])	0.84	1.67
MD07G1007400	K23260	scopoletin glucosyltransferase [EC:2.4.1.128]	0.77	1.74
MD05G1306500	K00430	Peroxidase ([EC:1.11.1.7])	7.55	6.45
MD05G1085800	K23260	Scopoletin glucosyltransferase ([EC:2.4.1.128])	4.38	4.59
MD03G1050900	K00487	Trans-cinnamate 4-monooxygenase [EC:1.14.14.91]	0.48	0.84
MD02G1242200	K05349	Beta-glucosidase ([EC:3.2.1.21])	2.99	2.65
MD02G1073400	K00588	Caffeoyl-CoA O-methyltransferase [EC:2.1.1.104]	0.92	0.85
MD00G1221400	K00487	Trans-cinnamate 4-monooxygenase ([EC:1.14.14.91])	0.44	0.80
MD00G1046200	K23260	scopoletin glucosyltransferase [EC:2.4.1.128]	1.66	4.13
MD05G1069000	K00430	Peroxidase [EC:1.11.1.7]	1.66	1.52
MD17G1265200	K00430	Peroxidase ([EC:1.11.1.7])	5.00	0
MD01G1229000	K13066	Caffeic acid 3-O-methyltransferase ([EC:2.1.1.68 2.1.1.4])	5.23	0
MD03G1110800	K13065	Shikimate O-hydroxycinnamoyltransferase [EC:2.3.1.133]	0	3.64

The KEGG results showed many DEGs with the annotated functions of plant-pathogen recognition, protein processing in the endoplasmic reticulum, GSH metabolism, endocytosis, MAPK signaling pathway, plant hormone signal transduction, and glycolysis/gluconeogenesis (Table S2). Among them, many DEGs were assigned to endocytosis and protein processing in the endoplasmic reticulum pathway after *Fpmd* MR5 inoculation (Fig. S9). These results strongly suggest that endocytosis and endosomal trafficking regulate the proteins targeted to the plasma membrane in response to pathogen attacks [30]. A group of up-regulated DEGs was assigned to the GSH metabolism pathway, such as *glutamate--cysteine ligase* (MD06G1082300), *GSH reductase* (MD04G1004900), *GSH POD* (MD06G1081300), *L-ascorbate POD* (MD12G1125600), and *GSH S-transferase* (MD05G1210700 and MD10G1172400), which potentially prevent oxidative damage from accumulated ROS [12] (Table S6). Multiple DEGs were up-regulated in the glycolysis/gluconeogenesis pathway (Table S7), such as *aldose 1-epimerase* (MD06G1189900), *6-phosphofructokinase 1* (MD08G1109700 and MD15G1089400), *fructose-bisphosphate aldolase, class I* (MD12G1018700), *glyceraldehyde 3-phosphate dehydrogenase* (MD17G1017300), *pyruvate kinase* (MD11G1001600), *pyruvate decarboxylase* (MD12G1173200, MD12G1172500, and MD12G1172400), *alcohol dehydrogenase* (NADP+) (MD00G1141700), and *alcohol dehydrogenase class-P* (MD05G1014600, MD05G1013200, and MD05G1013400).

Multiple DEGs with annotation functions in the plant hormone signal transduction pathway were identified from the *Fpmd* MR5 infected tissues. The biological functions of the proteins encoded by these DEGs in the ET and JA biosynthesis and signaling may not be readily categorized or highlighted by the KEGG Pathway Analysis (Table S8). Examples include JAZ (*asmonate ZIM domain-containing protein*: MD13G1127100 and MD16G1127400) and ERF (*ethylene-responsive TF 1*: MD04G1228800). Four auxin-responsive proteins (MD10G1176400, MD00G1016500, MD15G1169100, and MD12G1241700) were primarily up-regulated in response to *Fpmd* MR5 infection. Two DEG encoding *Gibberellin 2-beta-dioxygenase* (MD13G1148400 and MD10G1194100) showed up-regulated expression patterns.

Multiple DEGs with annotation were up-regulated in the plant-pathogen recognition and MAPK signaling pathways in the *Fpmd* MR5 infected group (Table S9-10), e.g., *calcium-dependent protein kinase* (MD03G1165100), *calcium-binding protein* (MD17G1257900, MD16G1152300, MD09G1013600, MD13G1151300, and MD10G1150400), *heat shock protein* (HSP) *90 kDa beta* (MD01G1208700, MD07G1279200, and MD07G1279100), *WRKY TF* (MD03G1057400, MD07G1280300, and MD07G1131400), *P-type Cu*^*+*^ *transporter* (MD14G1102600), *ethylene-responsive TF 1* (MD04G1228800), calmodulin (CaM) (MD16G1152300 and MD13G1151300), and *serine/threonine-protein kinase OXI1* (MD10G1154200). Several DEGs associated with PR proteins showed up-regulation (Table 3), including *glucan endo-1,3-beta-glucosidase* (MD07G1198700 and MD15G1031700), *endochitinase* (MD04G1048000), four *thaumatin-like proteins*, six PR *proteins*, two *pectin methylesterase inhibitors*, and *polygalacturonase*. *Pectin esterase inhibitor* and *polygalacturonase* encoding genes were more highly expressed in the *Fpmd* MR5 infected roots than the mock-inoculated roots. Xiang et al. [3] found that increases in plant secondary metabolism DEGs (UDP-glycosyltransferase and cytochromes P450) encode PRs in the defense response. We observed similar phenomenon results. Some DEGs related to stress responses and oxidative metabolism were also detected in this study, such as seventeen *GSH S-transferases*, one *monodehydroascorbate reductase*, *L-ascorbate POD*, and five *“heat shock 70 kDa proteins”*, indicating that these genes may have been activated in the early infection stage to resist *Fpmd* MR5 infection. A similar result was observed by Shin et al. [14]. The results indicated that plant-pathogen recognition, hormone signaling, and biosynthesis and transportation of secondary metabolites and PR proteins were up-regulated in the apple roots in response to the *Fpmd* MR5 infection.

**Table 3:** Identified DEGs functioning in other aspects of defense response

GeneName	Function	log2FoldChange	
		T2vsT1	M2vsM1
MD06G1164400	Cytochrome P450	5.58	6.17
MD06G1164300	Cytochrome P450	6.73	6.22
MD15G1255700	Cytochrome P450	4.77	4.27
MD03G1281500	Cytochrome P450	4.41	4.95
MD13G1148400	Gibberellin 2-beta-dioxygenase	2.43	1.71
MD10G1194100	Gibberellin 2-beta-dioxygenase	2.18	3.63
MD09G1065000	UDP-glycosyltransferase	5.09	6.01
MD04G1019600	UDP-glycosyltransferase	4.12	3.77
MD09G1141500	UDP-glycosyltransferase	3.45	5.30
MD05G1246700	UDP-glycosyltransferase	4.39	7.01
MD07G1198700	Glucan endo-1,3-beta-glucosidase	1.55	2.16
MD15G1031700	Glucan endo-1,3-beta-glucosidase	3.48	3.52
MD16G1161400	Pathogenesis-related protein	3.51	3.21
MD16G1026200	Pathogenesis-related protein	4.90	2.60
MD08G1036400	Pathogenesis-related protein	4.54	4.41
MD13G1023000	Pathogenesis-related protein	4.14	4.21
MD16G1026000	Pathogenesis-related protein	5.09	2.50
MD09G1247200	Pathogenesis-related protein	7.22	2.44
MD04G1048000	endochitinase	1.59	3.07
MD08G1011900	thaumatin-like protein	2.48	4.20
MD02G1130400	thaumatin-like protein	2.01	3.09
MD08G1011700	thaumatin-like protein	2.52	2.89
MD08G1011600	thaumatin-like protein	2.20	2.57
MD02G1207900	pectin methylesterase inhibitors	5.74	4.02
MD07G1053300	pectin methylesterase inhibitors	4.25	4.22
MD14G1062500	Polygalacturonase	1.95	3.20
MD07G1011600	Polygalacturonase	1.48	4.88
MD12G1195100	Serine/threonine-protein phosphatase PP1	5.36	5.59
MD08G1072800	Endoglucanase	4.74	6.62
MD15G1031700	Endoglucanase	3.48	3.52
MD16G1192600	Heat shock 70 kDa protein	7.27	6.27
MD17G1226000	Heat shock 70 kDa protein	7.34	7.38
MD17G1226100	Heat shock 70 kDa protein	6.23	7.07
MD17G1225800	Heat shock 70 kDa protein	5.34	5.42
MD12G1125600	L-ascorbate peroxidase	6.52	7.95
MD17G1032700	Monodehydroascorbate reductase	2.00	2.84
MD17G1238500	Transcription factor bHLH36	5.58	7.50
MD13G1096700	MFS transporter, NNP family, nitrate/nitrite transporter	3.11	3.80
MD15G1357900	nitrate reductase (NAD(P)H)	1.54	4.09
MD03G1121900	alpha carbonic anhydrase 7-like	4.68	4.30
MD11G1140500	alpha carbonic anhydrase 7-like	3.40	3.72
MD03G1017300	receptor-like kinases	3.57	5.29
MD03G1272700	receptor-like kinases	1.19	2.76
MD06G1218800	wall-associated receptor kinase	5.72	4.73
MD07G1070400	wall-associated receptor kinase	3.23	2.89

### Selection of the core candidate genes related to apple root susceptibility to *Fpmd* MR5

Based on the results of the RNA-Seq transcriptome analysis, we screened the up-regulated genes of the M.26 and M9T337 rootstock roots in response to the *Fpmd* MR5 infection (Fig. S10A-C). We used the results of KEGG and GO to obtain the first set of candidates according to the FPKM ratio of M9T337 (replant tolerant rootstock)/M.26 (replant susceptible rootstock) > 1, including those involved in various plant defense responses, e.g., hormonal signaling, WRKY transcription factor, secondary metabolite biosynthesis (beta-glucosidase), and PR-related proteins (Table S11, Fig. 5). The second set of candidates was selected from the remaining DEGs based on the changes in expression levels (Table 3), such as the PR-related protein *endochitinase*. A total of 33 core candidates were selected. We then verified if these candidate DEGs represented key genes involved in the apple root responses to *Fpmd* MR5 infection.

### qRT-PCR validation of DEG expression patterns

**Fig. 7:**
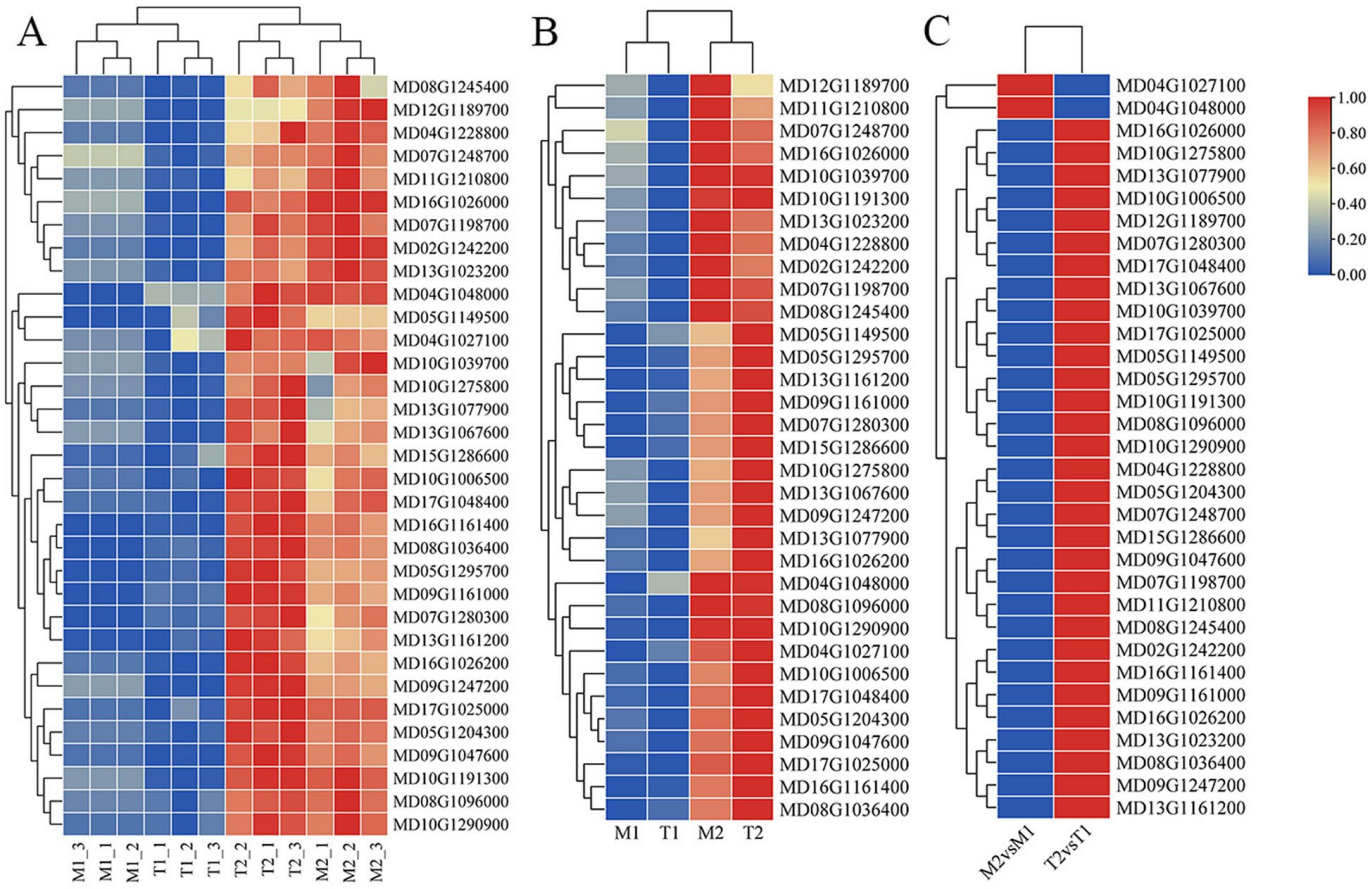
Validation of the expression patterns for genes selected from RNA-seq analysis by realtime qRT-PCR. Gene expression values were obtained by normalizing the values. GAPDH was used as aninternal control. **A** Differences in gene expression levels among different treatments. **B** Differences in mean gene expression levels among different treatments. **C** The qRT-PCR data ratio of up-regulated DEGs of M9T337 (T) or M.26 (M) dataset. The color intensity was proportional to the normalizing the values. Taxa relative abundances were log10-transformed, and the scale method (from zero to one) was used for the heatmap representation. Each treatment included three repetitions

RNA-Seq and qRT-PCR were used to quantify the expression levels of the candidate genes, and the results were consistent (Table S11, Fig. 7). The expression levels of MD13G1077900 (WRKY61), MD13G1067600 (WRKY3), MD10G1191300 (ERF31), MD05G1204300 (ERF1B), MD09G1047600 (WRI1), MD16G1026200 (MLP328), MD09G1247200 (PR10), and MD16G1026000 (MLP34) varied more than 2-fold between the M2vs.M1 and T2vs.T1 datasets, indicating that these genes may play a decisive role in apple root resistance to *Fpmd* MR5 infection.

## Discussion

Pathogenic fungi accumulate over time in the rhizosphere when apples are grown for many years in the same location [5]. The fungi adversely affect the growth and development of the root system and destroy root epidermal cells and cortical tissue, resulting in root tip necrosis, slow lateral root development, and a reduction or lack of functional root hairs [5, 4, 31]. The mechanism of the apple root response to ARD-related *P. ultimum* infection has been reported [1, 14]. However, the pathogens closely related to the occurrence of ARD in China are *Fusarium* spp. [3]. Thus, this study focused on *Fpmd* MR5 infection. The differential expression patterns of mock-inoculated/disease-inoculated and resistant/susceptible apple rootstocks were investigated to gain a better understanding of the molecular and physiological responses of the plant roots affected by ARD-related *Fpmd* MR5 and guide strategies to overcome the disease.

Many studies have found that the accumulation of ROS occurs in the early stage of plant-pathogen interaction. The strong antioxidant and radical scavengers ascorbate (AsA) and GSH can enhance the activity of the ROS scavenging system [14, 28]. Many DEGs related to AsA and GSH were up-regulated in this study, indicating that these genes may play a critical role in protecting apple root tissues from high levels of ROS due to *Fpmd* MR5 infection. Chen et al. [4] found that transcripts encoding cell wall degradation (i.e., pectate lyases, pectin methylesterase inhibitors (PMEI), and polygalacturonases (PG)) were up-regulated during *F. oxysporum* infection in bean. We found 2 PMEI/PG and 80 DEGs encoding for LRR proteins (data not shown). They were highly expressed proteins in apple seedlings infected with *Fpmd* MR5, suggesting that the cell wall is the plant’s first line of defense to limit the entry of pathogens [32]. Several genes involved in phenylalanine ammonia-lyase (PAL) pathways were also highly expressed. They regulate lignin accumulation and the formation of defensive structures in resistant plants in response to *Fpmd* MR5 infection [7]. In addition, we also found that two genes related to laccase synthesis (MD04G1142900 and MD04G1142300) were also significantly up-regulated. These results show that plants can defend themselves against pathogenic fungi by perceiving endogenous signals originating from their cell walls.

Most over-expressions of pathogenesis-related proteins (thaumatin-like protein, beta-glucosidase, or chitinase) protect plants against fungal pathogens [2, 3, 33]. PR proteins and hydrolytic enzymes, such as endo-1, 3-beta-glucosidase, and endochitinase (PR-3, − 4, − 8, and − 11) can disintegrate the cell wall of the necrotrophic pathogen and limit pathogen activity, growth, and spread [34]. Zhou et al. [32] found that *MdPR4* is a chitin-binding protein in apple vegetative tissues that may play an important role in defense activation in response to ARD-related *Fusarium* spp. pathogens. Xiang et al. [3] found that chitinase, β-1,3-glucanase, and UDP-glycosyltransferase may be crucial in the defense against *F. solani* infection. A similar phenomenon was observed in this study. A large number of PRs were activated after apple root infection with *Fpmd* MR5, and PR5 exhibited a more than 20-fold difference between the M2vs.M1 andT2 vs.T1 datasets, suggesting pathogenesis-related protein 5 and glucan endo-1,3-beta-glucosidase may be crucial in defense responses against *Fpmd* MR5. In addition, CaM-related DEGs were also induced, suggesting the involvement of the CaM-dependent signaling pathway (Table S9). We need to validate the function of these genes in plant infection to elucidate the defense mechanism of apple root against soilborne fungal pathogen infection.

Plant defense response signals may be amplified by the generation of secondary signal molecules, such as SA, ET, and JA, which play an important role in defense signaling networks [35]. Our results confirmed that hormones were crucial in signaling pathways in apple roots defending against *Fpmd* MR5 infection. Many DEGs related to SA (regulatory protein NPR1), ET (8 ethylene-responsive TF and 4 ET-insensitive protein 3), and JA (four ZIM domain-containing proteins) biosynthesis and signal transduction were activated after the *Fpmd* MR5 infection of apple roots. A strong induction of auxin-responsive proteins and IAA-encoding genes was also observed (Table S8, 11). This result is consistent with a previous study that found *F. oxysporum* infection activated the transcription of auxin-related genes, enhancing auxin biosynthesis [4]. It is known that JA and ET regulate the defense against several necrotrophic pathogens [2, 35], and some ET responsive proteins such as ET-insensitive protein-2 genes, are involved in the response to *F. oxysporum* infection in banana [36]. Hence, their activation/expression suggests that these genes (ET-insensitive protein 3) might be involved in responses to *Fpmd* MR5 infection and should be investigated further. ET can also induce the activation and accumulation of PR proteins and antimicrobial peptides, including glucanase, chitinase, and osmotin [2, 37]. JA has also been shown to regulate plant resistance to necrotrophic pathogens, such as *F. oxysporum* and *Fusarium fujikuroi* [38, 39]. We also observed the activation of the MAPK cascade. This pathway was significantly enriched (Table S10). It is well known that the MAPK-mediated signal transduction cascade is essential during defense activation in response to pathogenic pressure [40]. Overall, our results suggest an efficient and coordinated activation of several molecular components is needed for a successful resistance response, including early signal transduction (MAPKs), biosynthesis of defense hormones (IAA, ET, and JA), and transporters (ABC transporter family protein), similar to what has been reported in rice and banana [13, 38].

TFs are crucial components of plant defense, the coordination of hormone signal interactions, the regulation of cell wall component remodeling, and many cell physiological processes [35]. Members of the WRKY, AP2/ERF, NAC, MYB, and MYC/bHLH families have been shown to regulate defense-related gene expressions [28]. Among them, ERFs are responsive to pathogen-induced and exogenously applied ET and JA and regulate downstream PR genes [2, 37]. WRKY (containing the WRKYQK protein domain) can regulate several signaling networks, including MAPKs, histone deacetylases, chitin, and phytohormones (ABA and ET) [35, 41], and are involved in the biosynthesis of secondary metabolites [42], suggesting that WRKY and ERF transcription factors are crucial in the interaction between plants and pathogens [4, 28, 38]. This phenomenon has been observed in many studies. For example, Wang et al. [43] found that the overexpression of *VqERF112*, *VqERF114*, and *VqERF072* in transgenic *Arabidopsis* enhanced the resistance to *Pseudomonas syringae* pv. *tomato* DC3000 and *Botrytis cinerea* and increased the expression of the SA/JA/ET signaling-related genes. Li et al. [44] found that the overexpression of *CsWRKY50* in cucumber (*Cucumis sativus*) enhanced plant resistance to the fungal pathogen *Psilocybe cubensis* and up-regulated the transcript levels of several phytohormone-related (SA- and JA-responsive genes and SA biosynthesis genes) defense genes. Davis et al., [45] reported SA and JA induction of chitinase (PR3) in pine seedlings inoculated with *F. subglutinans* f. sp. *pini*, suggesting a potential role of PR proteins in pine defense. Similarly, Carrasco et al. [46] found a synchronized increase between the induction of PR5 and ET in *Pinus radiata* seedlings inoculated with *Fusarium circinatum*. The above studies show that multiple transcription factors induce certain PR proteins involved in the immune response to pathogenic fungi. In the present study, we detected up-regulation of the WRKY/ERF TF and PRs genes (particular PR-10) by RT-qPCR after apple root infection with *Fpmd* MR5. Therefore, this study focused on exploring the regulation mechanism of the WRKY and ERF transcription factors in the PR protein in apple root. The production and transportation of secondary metabolites are crucial to infection resistance and repair of damaged plant tissues, and these secondary metabolites have direct antibacterial effects (pathogen membrane disruption and pathogen protein/enzyme alteration) or indirect effects on cell wall enhancement (e.g., lignification, callose deposition) or act as signaling molecules for defense responses [47]. For example, proteins of the cytochrome P450 family can control the biosynthesis of diverse signaling molecules, and secondary metabolites are involved in the stress response of plants [3, 33]. Zabala et al. [48] found that many secondary metabolites derived from multiple branches of the phenylpropanoid pathway, including lignins, isoflavonoid-phytoalexins, other phenolic compounds, and SA are instrumental in the plant’s ability to mount successful defenses to invading pathogens. Likewise, many DEGs associated with the cytochrome P450 family and phenylpropanoid biosynthesis pathway were activated in this study after the apple roots were infected with *Fpmd* MR5. The protein function genes included HSPs that are crucial for dealing with biotic stress [31]. Here, the *HSP70* and *HSP90* genes were up-regulated after the apple root infection with *Fpmd* MR5, highlighting the importance of these genes in maintaining metabolism and growth.

It was previously reported that alterations in carbohydrate metabolism could occur during pathogen stress in plants [49]. Erayman et al. [50] observed a strong interaction between *Fusarium graminearum* and wheat, mainly involving the starch and sucrose metabolism, purine metabolism, and glycolysis/gluconeogenesis pathways. Glycolysis/gluconeogenesis is a catabolic anaerobic pathway that oxidizes hexoses to generate ATP, reducing agents and pyruvate and producing building blocks for anabolism [51]. In this study, aldose 1-epimerase, fructose-bisphosphate aldolase, glyceraldehyde 3-phosphate dehydrogenase (phosphorylating), enolase, pyruvate decarboxylase, phosphoenolpyruvate carboxykinase, and alcohol dehydrogenase were identified as significantly up-regulated genes (Table S7), providing evidence that the glycolysis/gluconeogenesis pathway is involved in the infection response. Similarly, glycolysis/gluconeogenesis metabolism has also been involved in the response to *Rhizoctonia solani* infection, and susceptible cultivars defend against *R. solani* infection by increasing the abundance of glycolysis-related proteins; thus, more ATP is required [52].

Nitrogen and carbon sources are necessary for living organisms and need to be obtained from the host plants by pathogens [53]. It is well known that phosphoenolpyruvate carboxylase (PEPC), NADP-malic enzyme (NADP-ME), and NAD-MDH form a metabolic cycle under stress in C3 plants [54]. The enhanced activities of PEPC and/or NADP-ME enable C3 plants to cope with environmental stress. Similarly, Miyao and Fukayama [55] found that the overexpression of PEPC in plants increased the activities of NADP-ME and NADP isocitrate and the contents of pyruvate, glutamate, and aspartate. Pyruvate enters the citrate cycle (TCA cycle) due to the combined actions of PEPC, MDH, and NADP-ME, indicating that the TCA cycle and the subsequent amino acid synthesis were enhanced in these plants. In addition, NADPH produced by the TCA cycle contributes to redox homeostasis and plant defense against pathogens [56]. Under stress conditions, PEPC improves the whole-plant carbon gain by refixing the internally released CO_2_, which is crucial for plant defense [57]. In this study, many DEGs were enriched in the carbon metabolism, the alanine, aspartate, and glutamate metabolism, pyruvate metabolism, and the TCA cycle pathways in the root transcriptome of the resistant M9T337. The upregulation of the major facilitator superfamily (MFS) transporter, nitrate/nitrite transporter (NNP), nitrate reductase (NAD(P)H), and alpha carbonic anhydrase 7-like indicate the role of nitrogen mobilization. These genes are closely related to the synthesis of metabolites (glutamate and glutamine) (Table 3). Glutamate is required for the synthesis of GSH and is required in the carbon metabolism and signaling pathways in plants. Exogenous glutamate (10 mM) has been shown to induce systemic disease resistance in rice [58]. These results suggest that apple roots might rapidly reprogram their carbon and nitrogen metabolisms to provide energy and metabolic sources for defense after infection with *Fpmd* MR5.

## Conclusions

**Fig. 8:**
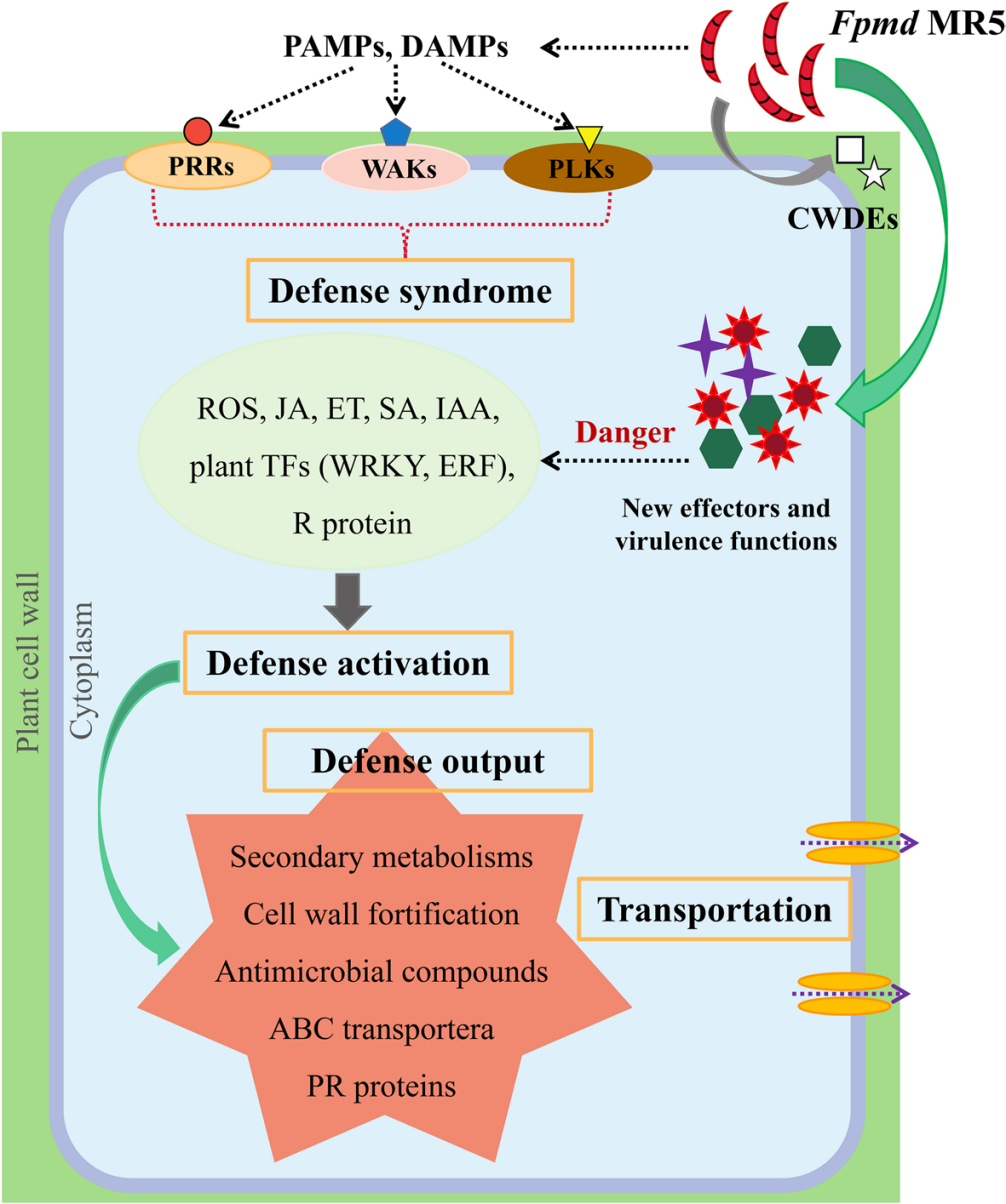
The illustration of molecular network underlying the defense response in apple root in response to the infection by *Fpmd* MR5. PRRs, RLKs, and WAKs located in the cell membrane of apple roots can recognize PAMPs/DAMPs to detect the presence of *Fpmd* MR5 and initiate the plant immune system (PTI) to activate defense responses, including phytohormone biosynthesis and/or ROS generation, as well as induction or repression of TFs (WRKY and ERF). Resistant R proteins in plants can also interact directly/indirectly with effector proteins secreted by *Fpmd* MR5 to initiate ETI responses. As a result of defense activation (biphenyl, spermidine), numerous antimicrobial compounds, antioxidant production (glutathione, flavonols, dihydroflavonols, anthocyanins), and pathogenesis-related proteins (endochitinasem, thaumatin-like protein, Glucan endo-1,3-beta-glucosidase) are produced, and cell walls are strengthened (laccase, lignin). These antimicrobial components are delivered to infection sites by various transporters to limit the adverse effects of the pathogen

We performed root xylem transcriptome analysis of resistant M9T337 and susceptible M.26 rootstocks using RNA-Seq, revealing for the first time the dynamics of genome-wide defense responses in apple root tissues to *Fpmd* MR5 infection (Fig. 8). The biosynthesis and signaling of several plant hormones including ethylene, jasmonate and salicylic acid, lignin biosynthesis, ROS regulation by glutathiones participated in defense response to *Fpmd* MR5 infection. The production and accumulation of secondary metabolites was the main defense response in apple root. Additionally, genes encoding the biosynthesis of PRs such as beta-glucosidase or chitinase and several ERFs (ERF3, ERF1B, WRI1) and WRKYs (WRKY61 and WRKY3) involved in the synthesis of phytohormones and secondary metabolites were strongly induced genes by *Fpmd* MR5, and the expression of these genes played an important role in the apple defense against pathogenic infection. Future work should characterize the functions of the selected candidate DEGs involved in apple-*Fpmd* MR5 interactions (Table S11) and clarify their specific roles in plant defense mechanisms. The results could provide insights into the detailed regulatory mechanisms of plant diseases and guide the development of new strategies for controlling ARD-related pathogens.

## Supplementary Information

**Additional file 1.** Supplement

**Additional file 2:** **Supplementary Figures.**

## Data Availability

*Fpmd* MR5 was deposited at China General Microbiological Culture Collection Center (CGMCC) under the accession number CGMCC No. 22426. The *Fpmd* MR5 whole genome data presented in this paper have been deposited in GenBank under the accession number JAFHKW000000000.1 (https://www.ncbi.nlm.nih.gov/Traces/wgs/ ?val = JAFHKW01) (BioProject: PRJNA698749, BioSample: SAMN17735672). The raw sequence data are available in the NCBI Gene Expression Omnibus (GEO) repository. The accession number is GSE171688 (BioProject: PRJNA720576, SRA: SRP313987 (https://www.ncbi.nlm.nih.gov/geo/query/acc.cgi?acc=GSE171688)). *Malus* × *robusta* (CarriŠre) Rehder has been deposited in National Specimen information infrastructure (NSII) (http://www.nsii.org.cn/2017/home-en.php). The deposition number is SDF1003092. All data supporting the conclusions of this article are included in the article and its additional files.

## Abbreviations

*Fpmd* MR5: *Fusarium proliferatum* f. sp. *malus domestica*
PRRs: Pattern recognition receptors
PAMPs: Pathogen-associated molecular patterns
DAMPs: Damage-associated molecular patterns
PTI: PAMP-Triggered immunity
ETI: Effector-Triggered immunity
RLKs: Receptor-like kinases
WAKs: Wall-associated receptor kinases
ROS: Reactive oxygen species
ABC Transporter: ATP-binding cassette transporter
CWDEs: Cell wall degrading enzymes
ABA: Abscisic acid

## References

[1] Shin S, Lv J, Fazio G, Mazzola M, Zhu Y (2014). Transcriptional regulation of ethylene and jasmonate mediated defense response in apple (*Malus domestica*) root during *Pythium ultimum* infection. Hortic Res.

[2] Zhu Y, Fazio G, Mazzola M (2014). Elucidating the molecular responses of apple rootstock resistant to ARD pathogens: challenges and opportunities for development of genomics-assisted breeding tools. Hortic Res..

[3] Xiang L, Wang M, Pan F (2021). Transcriptome analysis *Malus domestica* ‘M9T337’root molecular responses to *fusarium solani* infection. Physiol Mol Plant Pathol.

[4] Chen L, Wu Q, He W, He T, Wu Q, Miao Y (2019). Combined de novo transcriptome and metabolome analysis of common bean response to *Fusarium oxysporum* f sp *phaseoli* infection. Int J Mol Sci.

[5] Mazzola M, Brown J, Zhao X, Izzo AD, Fazio G (2019). Interaction of brassicaceous seed meal and apple rootstock on recovery of *Pythium* spp. and *Pratylenchus penetrans* from roots grown in replant soils. Plant Dis.

[6] Johnson WC (2000). Methods and results of screening for disease-and insect-resistant apple rootstocks. Compact Fruit Tree.

[7] Tan G, Liu K, Kang J (2015). Transcriptome analysis of the compatible interaction of tomato with *Verticillium dahliae* using RNA-sequencing. Front Plant Sci.

[8] Erktan A, McCormack ML, Roumet C. Frontiers in root ecology: recent advances and future challenges. Plant and Soil. 2018;424(1):1–9.

[9] De Coninck B, Timmermans P, Vos C, Cammue BP, Kazan K (2015). What lies beneath: belowground defense strategies in plants. Trends Plant Sci.

[10] Glazebrook J (2005). Contrasting mechanisms of defense against biotrophic and necrotrophic pathogens. Annu Rev Phytopathol.

[11] Guo S, Zuo Y, Zhang Y (2017). Large-scale transcriptome comparison of sunflower genes responsive to *Verticillium dahliae*. BMC Genomics.

[12] Li C, Shao J, Wang Y (2013). Analysis of banana transcriptome and global gene expression profiles in banana roots in response to infection by race 1 and tropical race 4 of *fusarium oxysporum* f. sp. *cubense*. Bmc. Genomics..

[13] Xiang L, Wang M, Huang J, Jiang W, Yan Z, Chen X, et al. *MdWRKY74* is involved in resistance response to apple replant disease. Plant Growth Regul. 2022;96(1):145–56.

[14] Shin S, Zheng P, Fazio G, Mazzola M, Main D, Zhu Y (2016). Transcriptome changes specifically associated with apple (*Malus domestica*) root defense response during *Pythium ultimum* infection. Physiol Mol Plant Pathol.

[15] Duan Y, Jiang W, Zhang R, Chen R, Chen X, Yin C, et al. Discovery of *fusarium proliferatum* f. sp. *malus domestica* causing apple replant disease in China. Plant Dis. 2022. 10.1094/PDIS-12-21-2802-RE. 10.1094/PDIS-12-21-2802-RE 35306841

[16] Rehder A (1920). New species, varieties and combinations from the herbarium and the collections of the arnold arboretum (continued). J Arnold Arbor.

[17] Phipps JB, Robertson KR, Smith PG, Rohrer JR (1990). A checklist of the subfamily Maloideae (Rosaceae). Can J Bot.

[18] Parker ML, Hoyt T, Clark B. Evaluating apple replant strategies in the southeastern united states. Acta Horticulturae. 2014;1058(1058):645–50.

[19] Balbín-Suárez A, Lucas M, Vetterlein D (2020). Exploring microbial determinants of apple replant disease (ARD): a microhabitat approach under split-root design. FEMS Microbiol Ecol.

[20] Fradin EF, Zhang Z, Juarez Ayala JC (2009). Genetic dissection of *Verticillium* wilt resistance mediated by tomato Ve1. Plant Physiol.

[21] Bian X, Zhao Y, Xiao S, Yang H, Han Y, Zhang L (2021). Metabolome and transcriptome analysis reveals the molecular profiles underlying the ginseng response to rusty root symptoms. BMC Plant Biol.

[22] Sun P, Mao Y, Li G, Cao M, Kong F, Wang L, et al. Comparative transcriptome profiling of *Pyropia yezoensis* (Ueda) MS Hwang & HG Choi in response to temperature stresses. BMC Genomics. 2015;16(1):1–16. 10.1186/s12864-015-1586-1 PMC4470342 26081586

[23] Zhou F, Wang J, Chi X, Zhou X, Wang Z (2020). lncRNA TM4SF1-AS1 activates the PI3K/AKT signaling pathway and promotes the migration and invasion of lung cancer cells. Cancer Manag Res.

[24] Guo AY, Chen X, Gao G (2007). PlantTFDB: a comprehensive plant transcription factor database. Nucleic Acids Res.

[25] Kanehisa M, Furumichi M, Sato Y, Ishiguro-Watanabe M, Tanabe M (2022). KEGG: integrating viruses and cellular organisms. Nucleic Acids Res.

[26] Soltani R, Amini M, Mazaheri Moghaddam M, et al. LncRNA DLGAP1-AS2 overexpression associates with gastric tumorigenesis: a promising diagnostic and therapeutic target. Mol Biol Rep. 2022. 10.1007/s11033-021-07038-w. 10.1007/s11033-021-07038-w 34981339

[27] Xi L, Xu K, Qiao Y, Qu S, Zhang Z, Dai W (2011). Differential expression of ferritin genes in response to abiotic stresses and hormones in pear (*Pyrus pyrifolia*). Mol Biol Rep.

[28] Buscaill P, Rivas S (2014). Transcriptional control of plant defence responses. Curr Opin Plant Biol.

[29] Yazaki K (2006). ABC transporters involved in the transport of plant secondary metabolites. FEBS Lett.

[30] Wu G, Cui X, Chen H (2018). Dynamin-like proteins of endocytosis in plants are coopted by potyviruses to enhance virus infection. J Virol.

[31] Weiß S, Bartsch M, Winkelmann T (2017). Transcriptomic analysis of molecular responses in *Malus domestica* ‘M26’roots affected by apple replant disease. Plant Mol Biol.

[32] Houston K, Tucker MR, Chowdhury J, Shirley N, Little A (2016). The plant cell wall: a complex and dynamic structure as revealed by the responses of genes under stress conditions. Front Plant Sci.

[33] Zhou Z, Zhu Y, Tian Y (2021). *MdPR4*, a pathogenesis-related protein in apple, is involved in chitin recognition and resistance response to apple replant disease pathogens. J Plant Physiol.

[34] Broekaert W, Terras F, Cammue B. Induced and Preformed Antimicrobial Proteins. In: Slusarenko, A.J., Fraser, R.S.S., van Loon, L.C. (eds) Mechanisms of Resistance to Plant Diseases. Dordrecht: Springer; 2000. 10.1007/978-94-011-3937-3_11.

[35] Birkenbihl RP, Somssich IE (2011). Transcriptional plant responses critical for resistance towards necrotrophic pathogens. Front Plant Sci.

[36] Li CY, Deng GM, Yang J (2012). Transcriptome profiling of resistant and susceptible Cavendish banana roots following inoculation with *fusarium oxysporum* f. sp. *cubense* tropical race 4. BMC Genomics.

[37] Lorenzo O, Piqueras R, Sánchez-Serrano JJ, Solano R (2003). ETHYLENE RESPONSE FACTOR1 integrates signals from ethylene and jasmonate pathways in plant defense. Plant Cell.

[38] Matić S, Bagnaresi P, Biselli C (2016). Comparative transcriptome profiling of resistant and susceptible rice genotypes in response to the seedborne pathogen *fusarium fujikuroi*. BMC Genomics.

[39] Sun J, Zhang J, Fang H (2019). Comparative transcriptome analysis reveals resistance-related genes and pathways in *Musa acuminata* banana'Guijiao 9'in response to fusarium wilt. Plant Physiol Biochem.

[40] Pitzschke A, Schikora A, Hirt H (2009). MAPK cascade signalling networks in plant defence. Curr Opin Plant Biol.

[41] Phukan UJ, Jeena GS, Shukla RK (2016). WRKY transcription factors: molecular regulation and stress responses in plants. Front Plant Sci.

[42] Amato A, Cavallini E, Zenoni S (2017). A grapevine TTG2-like WRKY transcription factor is involved in regulating vacuolar transport and flavonoid biosynthesis. Front Plant Sci.

[43] Wang L, Liu W, Wang Y (2020). Heterologous expression of Chinese wild grapevine *VqERFs* in Arabidopsis thaliana enhance resistance to *pseudomonas syringae* pv. Tomato DC3000 and to *Botrytis cinerea*. Plant Sci.

[44] Li P, Song A, Gao C (2015). Chrysanthemum WRKY gene CmWRKY17 negatively regulates salt stress tolerance in transgenic chrysanthemum and Arabidopsis plants. Plant Cell Rep.

[45] Davis JM, Wu H, Cooke JE, Reed JM, Luce KS, Michler CH (2002). Pathogen challenge, salicylic acid, and jasmonic acid regulate expression of chitinase gene homologs in pine. Mol Plant-Microbe Interact.

[46] Carrasco A, Wegrzyn JL, Durán R (2017). Expression profiling in Pinus radiata infected with *fusarium circinatum*. Tree Genet Genomes.

[47] Pusztahelyi T, Holb IJ, Pócsi I (2015). Secondary metabolites in fungus-plant interactions. Front Plant Sci.

[48] Zabala G, Zou J, Tuteja J, Gonzalez DO, Clough SJ, Vodkin LO (2006). Transcriptome changes in the phenylpropanoid pathway of Glycine max in response to *pseudomonas syringae* infection. BMC Plant Biol.

[49] Gurkok T, Turktas M, Parmaksiz I, Unver T (2015). Transcriptome profiling of alkaloid biosynthesis in elicitor induced opium poppy. Plant Mol Biol Report.

[50] Erayman M, Turktas M, Akdogan G (2015). Transcriptome analysis of wheat inoculated with *fusarium graminearum*. Frontiers. Plant Sci.

[51] Plaxton WC (1996). The organization and regulation of plant glycolysis. Annu Rev Plant Biol.

[52] Ma H, Sheng C, Qiao L, Zhao H, Niu D (2020). A comparative proteomic approach to identify defence-related proteins between resistant and susceptible rice cultivars challenged with the fungal pathogen *Rhizoctonia solani*. Plant Growth Regul.

[53] De Yuan P, Xu XF, Hong WJ, et al. Transcriptome analysis of rice leaves in response to *Rhizoctonia solani* infection and reveals a novel regulatory mechanism. Plant Biotechnology Reports. 2020;14(5):559–73.

[54] Chojak-Koźniewska J, Kuźniak E, Linkiewicz A, Sowa S (2018). Primary carbon metabolism-related changes in cucumber exposed to single and sequential treatments with salt stress and bacterial infection. Plant Physiol Biochem.

[55] Miyao M, Fukayama H (2003). Metabolic consequences of overproduction of phosphoenolpyruvate carboxylase in C3 plants. Arch Biochem Biophys.

[56] Mhamdi A, Mauve C, Gouia H, Saindrenan P, Hodges M, Noctor G (2010). Cytosolic NADP-dependent isocitrate dehydrogenase contributes to redox homeostasis and the regulation of pathogen responses in Arabidopsis leaves. Plant Cell Environ.

[57] Kuźniak E, Kornas A, Kaźmierczak A (2016). Photosynthesis-related characteristics of the midrib and the interveinal lamina in leaves of the C3–CAM intermediate plant *Mesembryanthemum crystallinum*. Ann Bot.

[58] Kan CC, Chung TY, Wu HY, Juo YA, Hsieh MH (2017). Exogenous glutamate rapidly induces the expression of genes involved in metabolism and defense responses in rice roots. BMC Genomics.

