# Supplementary material for: Transcriptome changes associated with apple (Malus domestica) root defense response after Fusarium proliferatum f. sp. malus domestica infection
Source: BMC Genomics. 2022 Jul 2;23:484. doi: 10.1186/s12864-022-08721-3 (PMC9250749; doi:10.1186/s12864-022-08721-3)

**Transcriptome changes associated with apple (*Malus domestica*) root defense response after *Fusarium proliferatum* f. sp. *malus domestica* infection**

Yanan Duan, Shurui Ma, Xuesen Chen, Xiang Shen, Chengmiao Yin*, and Zhiquan Mao*

State Key Laboratory of Crop Biology/College of Horticultural Science and Engineering, Shandong Agricultural University, Tai’an 271018, Shandong, China

*Corresponding author at: Shandong Agricultural University, Daizong Road No.61, Tai’an 271018, Shandong, China. Tel.: +86 538 8241984/+86 538 8768246.

E-mail address: mzhiquan@sdau.edu.cn (Z. Mao); yinchengmiao@163.com.

Supplementary material


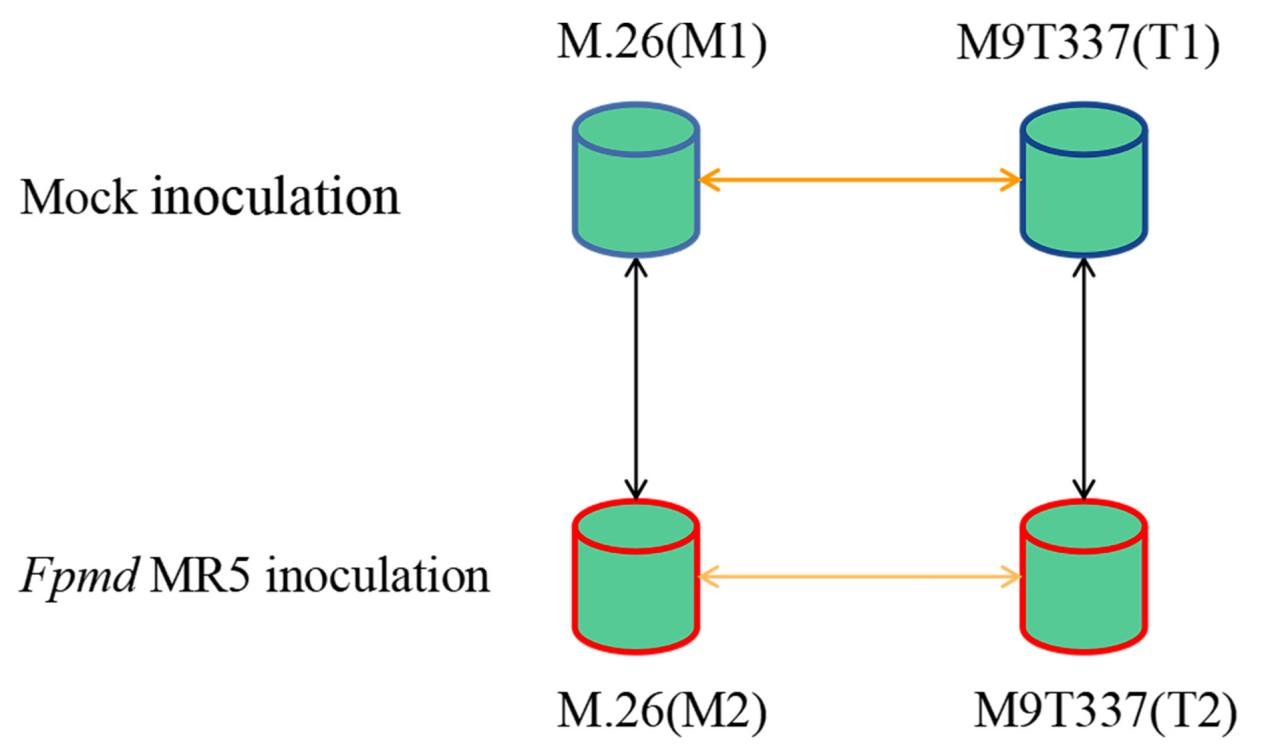
Figure S1. The illustrated experimental design and data analysis methods. Plant root tissues were collected for both mock inoculation and *Fpmd* MR5 inoculation at the designated time points. Two-way data analyses, as indicated by black and yellow lines, were performed for cross-examination on the specific transcriptomic changes associated with *Fpmd* MR5 infection in apple root tissue.

Figure S2. A: Sample gene expression distribution box plot. B: Violin plot of sample gene expression distribution. C: Sample gene expression distribution probability density distribution map.


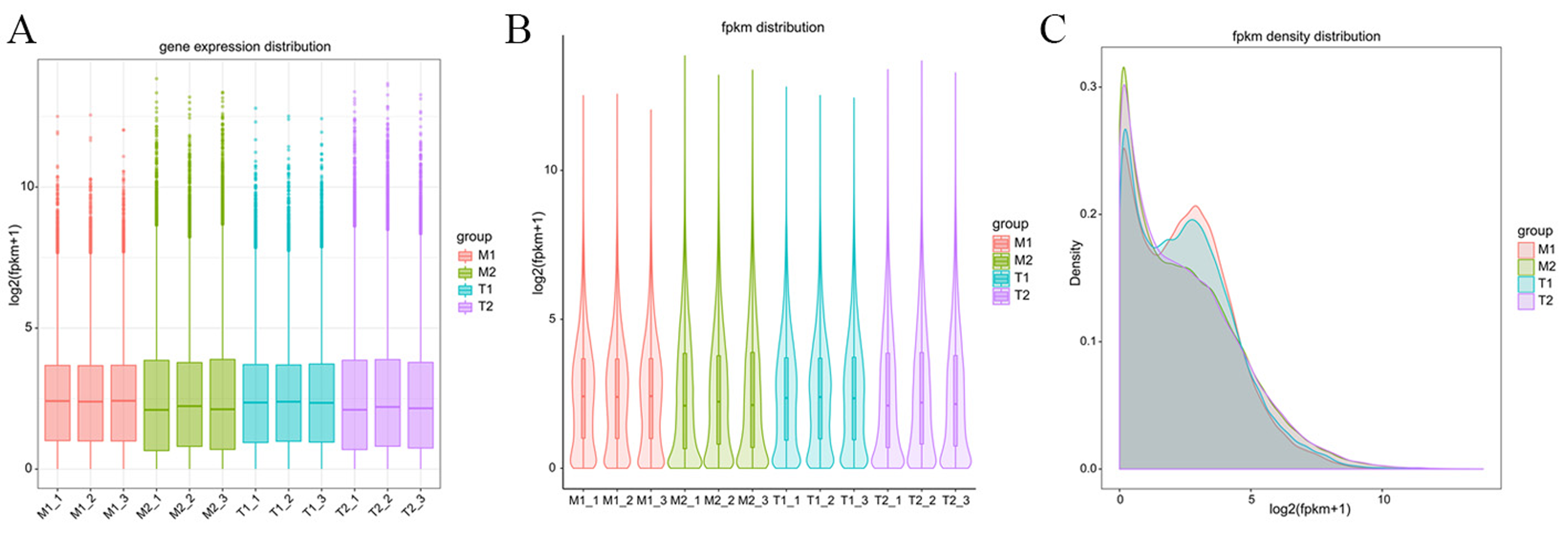


Figure S3. Analysis of differentially expressed genes (DEGs). A: DEGs Histogram. Blue and grey represent up-regulated and down-regulated differential genes, respectively, and the numbers on the bars represent the number of differential genes. B:DEGs Venn diagram. Different colors indicate different comparison combinations. C: DEGs Clustering heatmap. The abscissa is the sample name, and the ordinate is the normalized value of the differential gene FPKM. The redder the color, the higher the expression level, and the greener the expression level, the lower the expression level. D: DEGs Cluster Line Chart. The abscissa is the sample name, and the ordinate is the log2(fpkm+1) of the differential gene expression using the H-cluster method and the value after centering and correction. The gray line represents the relative correction of the genes in a cluster under the condition of no treatment. After gene expression level, the blue line represents the average value of the relative corrected gene expression level of all genes in this cluster under different treatment conditions.


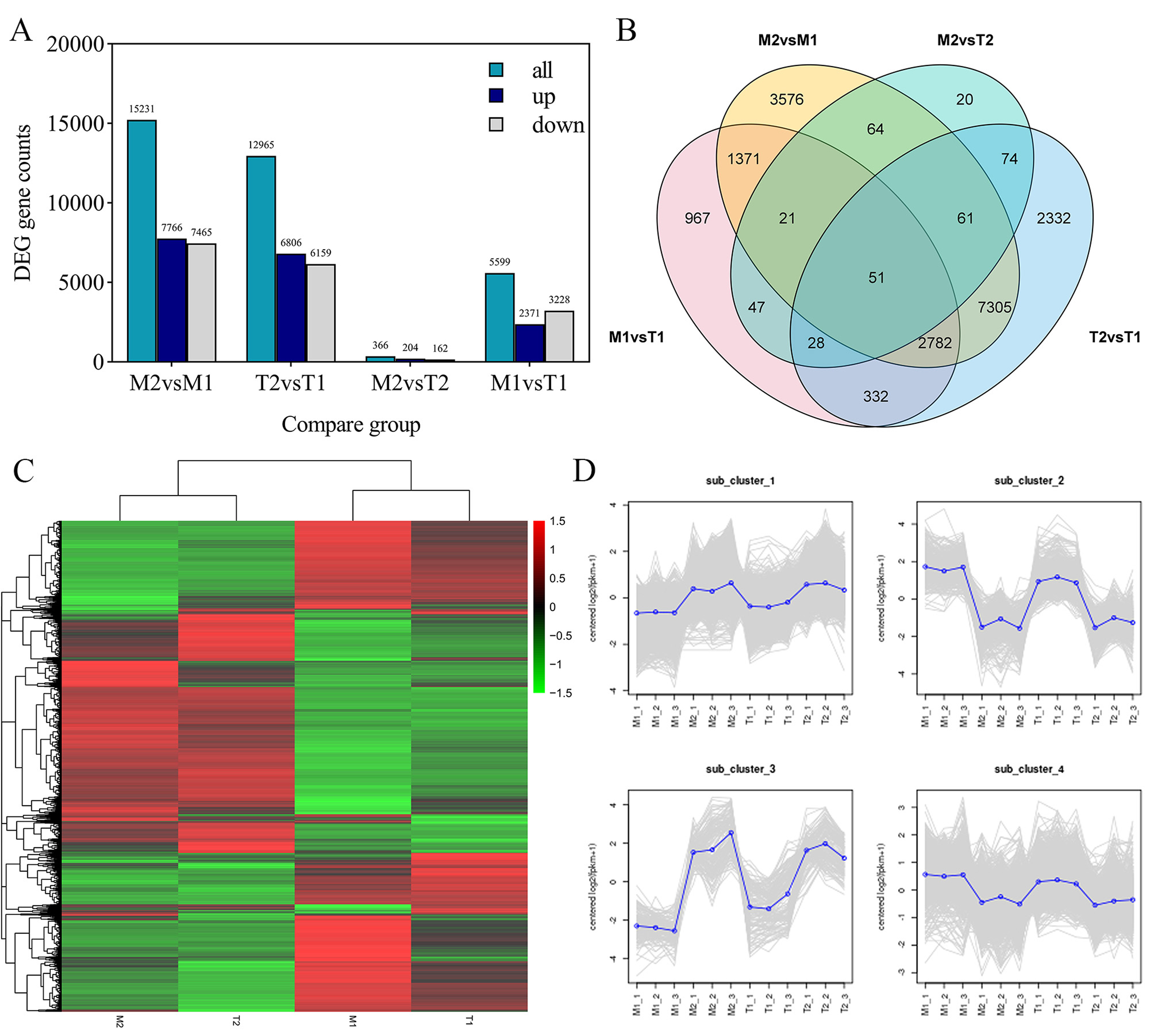


Figure S4. GO terms enriched in differentially expressed genes between mock inoculated and *Fpmd* MR5 infected (M2 and M1,T2 and T1). BP: biological process. CC: cellular component. MF: Molecular Function. Up-regulated genes are indicated by red, and down-regulated genes are indicated by green.


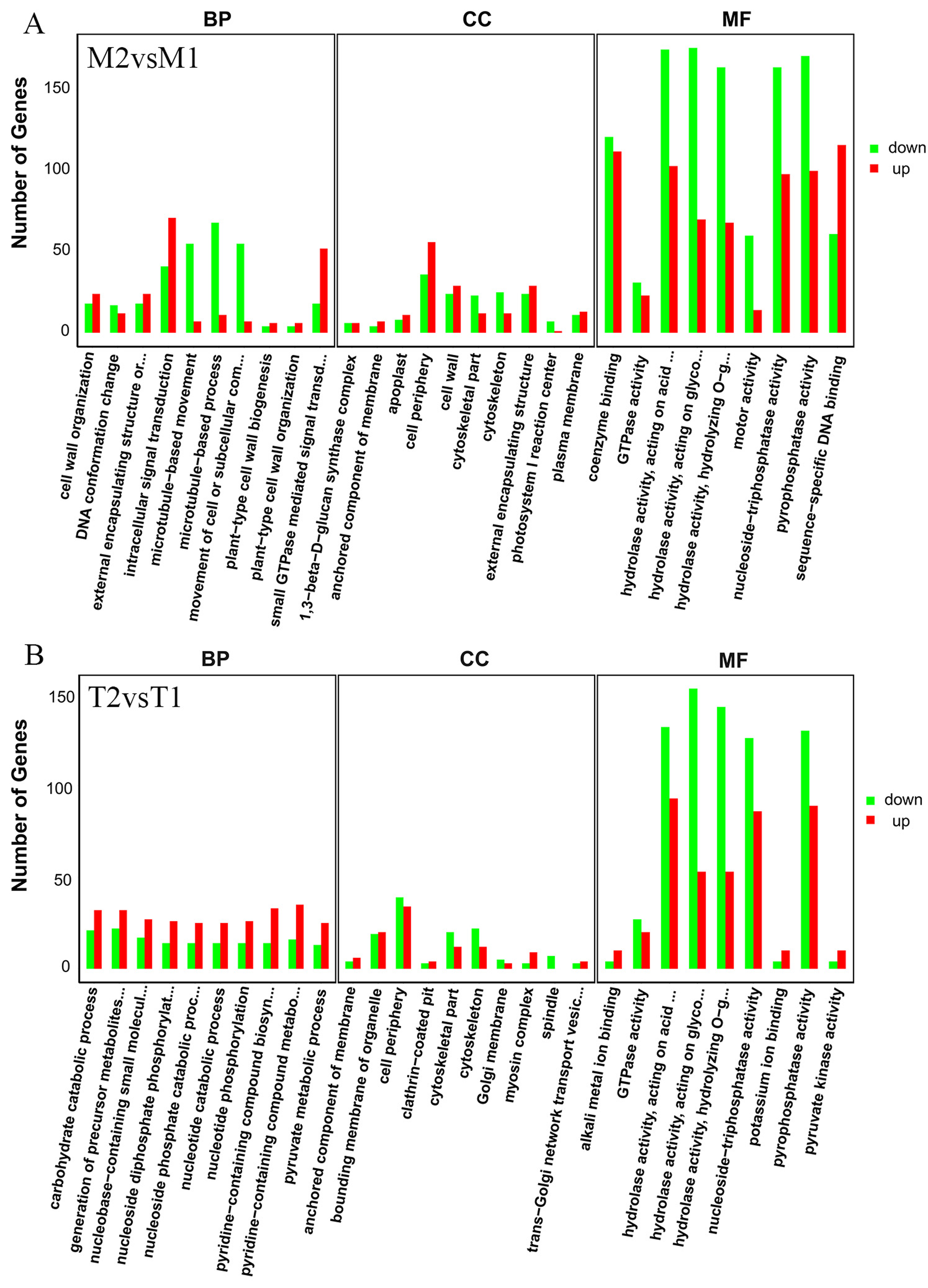


Figure S5. MapMan graphs of regulation in M2vs.M1 and T2vs.T1 datasets. The scale bar represents the log2FoldChange of the DEGs. Red and blue indicate upregulated and down regulated genes, respectively.


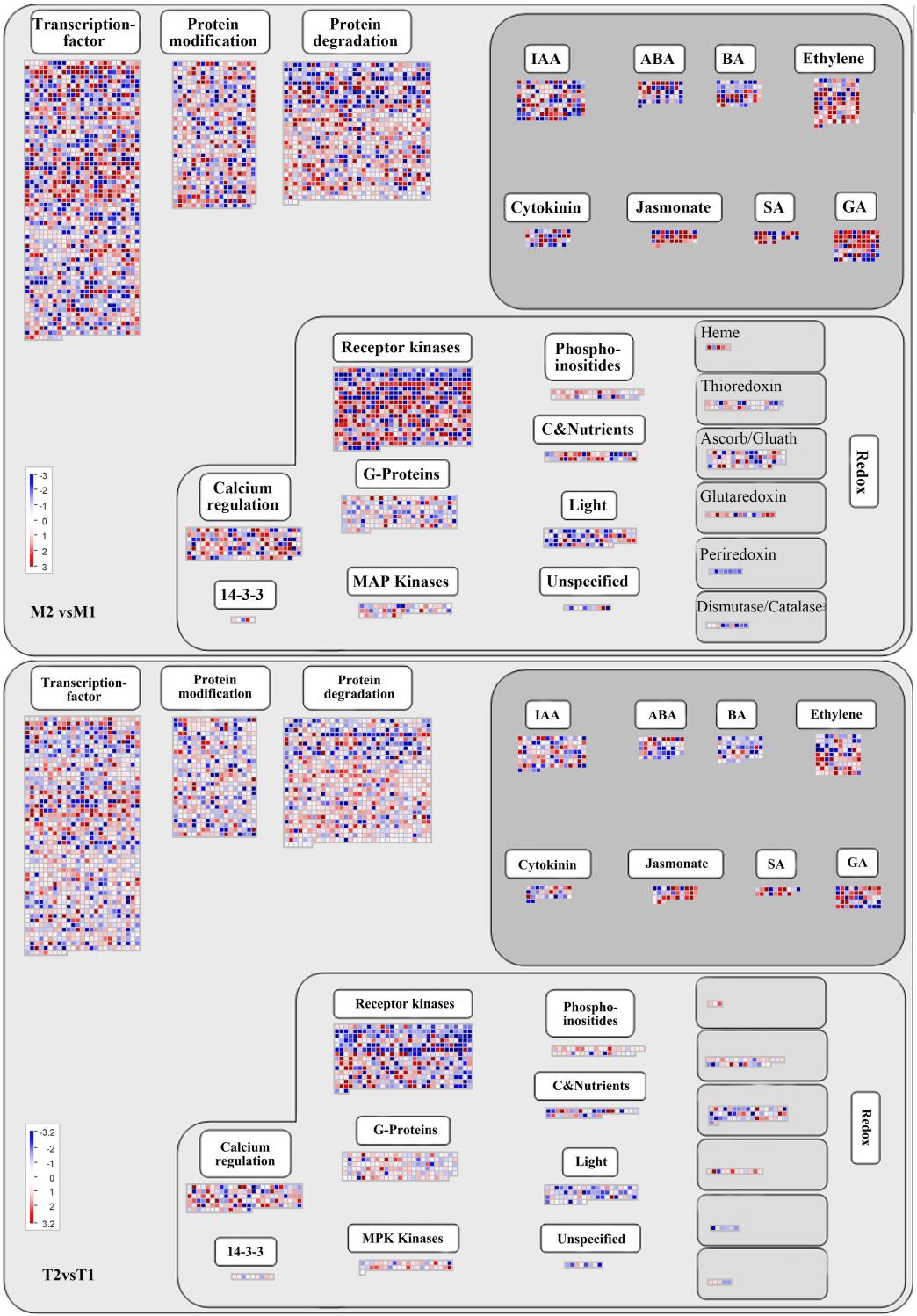


Figure S6. MapMan graphs of metabolism in M2vs.M1 and T2vs.T1 datasets. The scale bar represents the log2FoldChange of the DEGs. Red and blue indicate upregulated and down regulated genes, respectively.


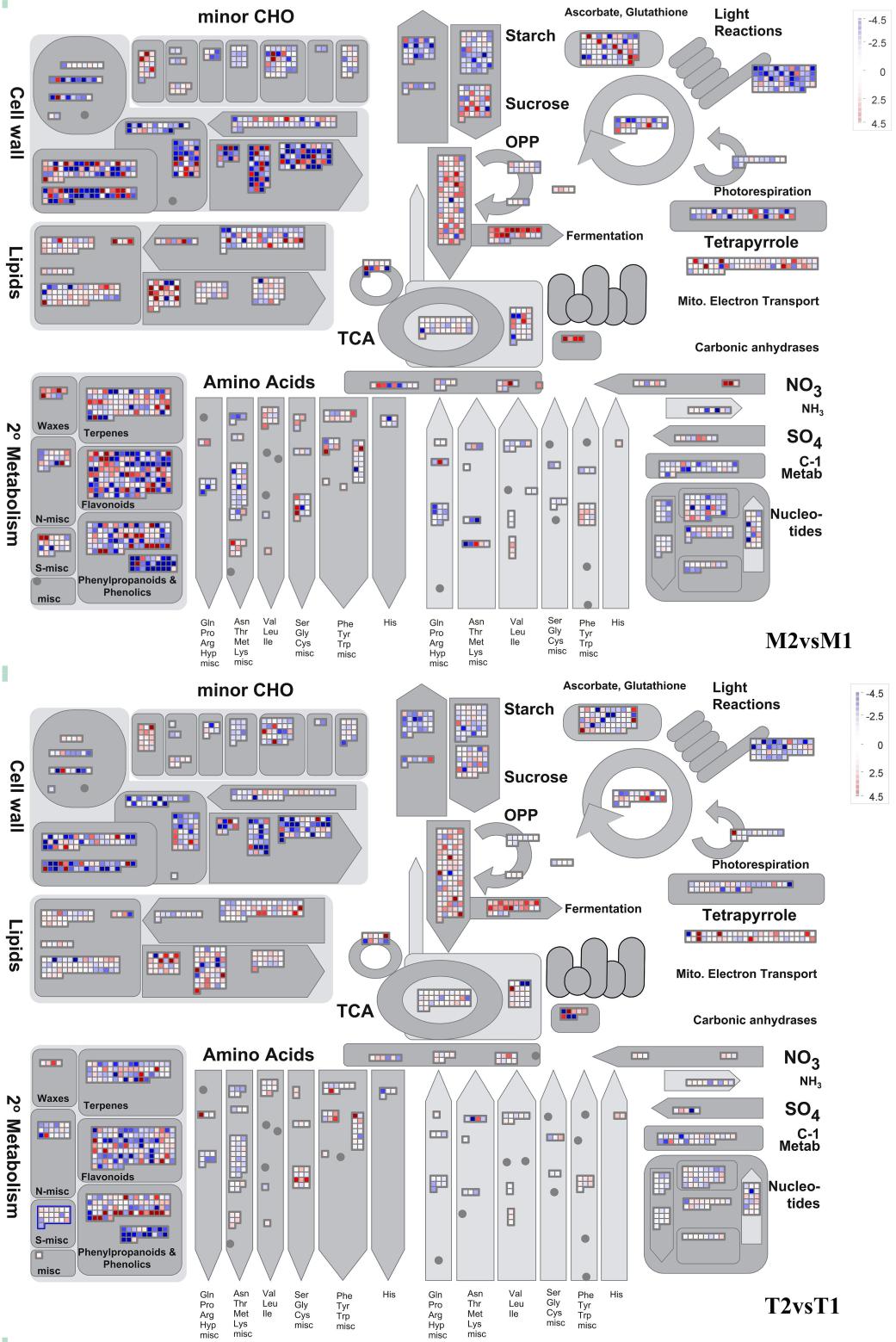


Figure S7. MapMan graphs of secondary metabolism in M2vs.M1 and T2vs.T1 datasets. The scale bar represents the log2FoldChange of the DEGs. Red and blue indicate upregulated and down regulated genes, respectively.


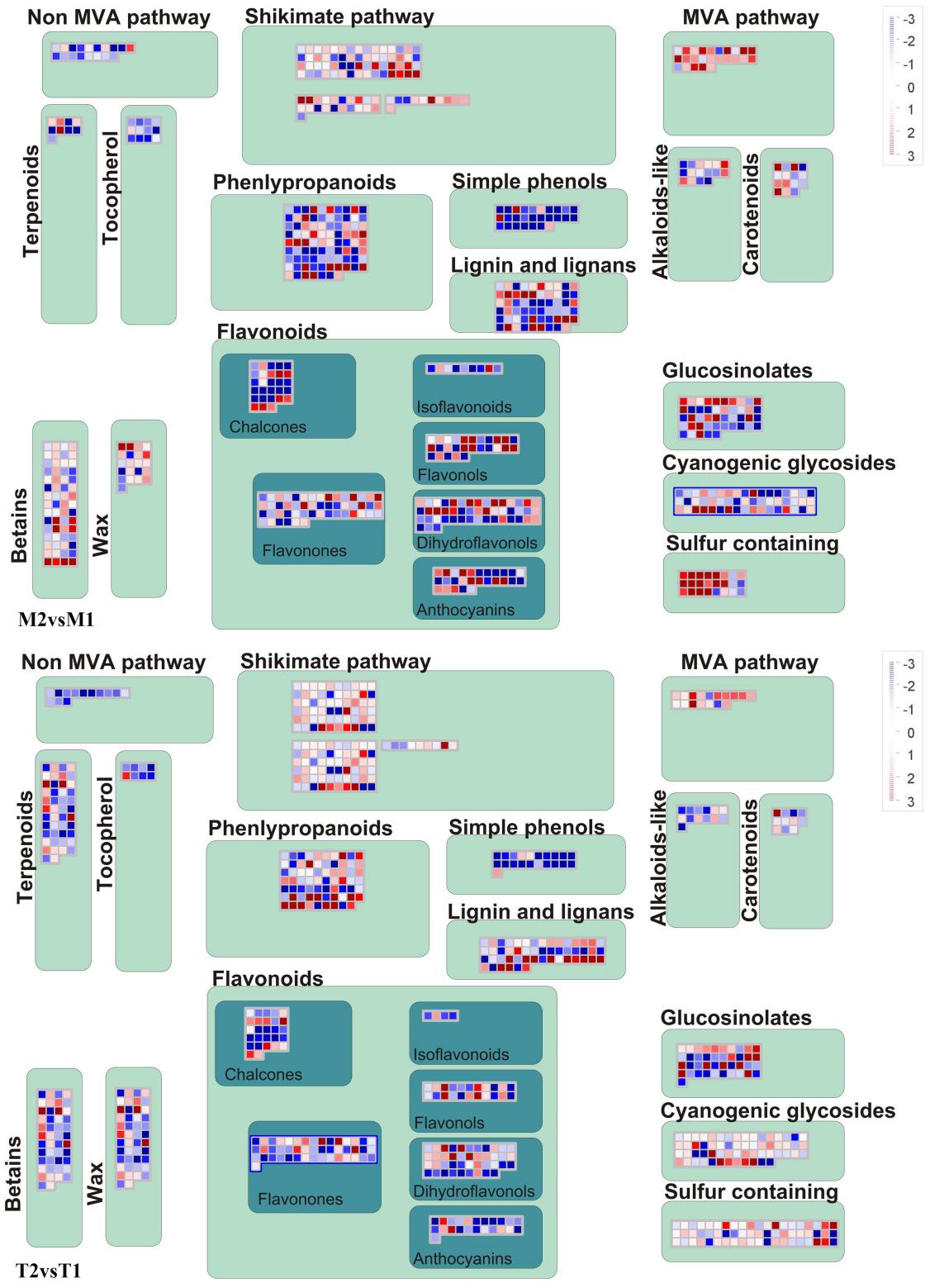


Figure S8. MapMan graphs of enzyme families in M2vs.M1 and T2vs.T1 datasets. The scale bar represents the log2FoldChange of the DEGs. Red and blue indicate upregulated and down regulated genes, respectively.


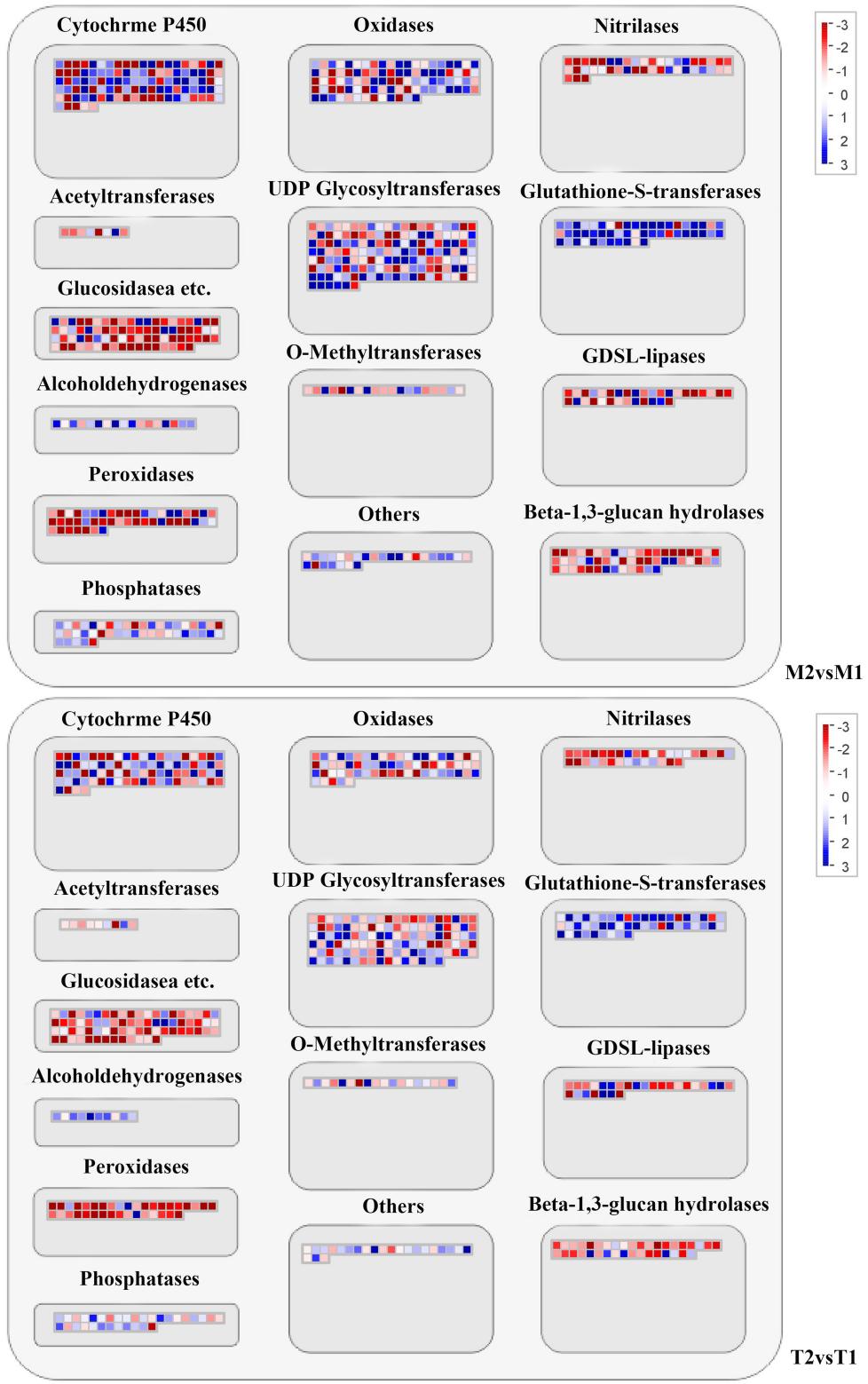


Figure S9. Endocytosis (mdm04144) and protein processing in endoplasmic reticulum (mdm04141) pathway. Based on pathway analysis, DEGs encoding specific proteins and mapped to Endocytosis (A) and protein processing in endoplasmic reticulum (B) pathway were highlighted in red.


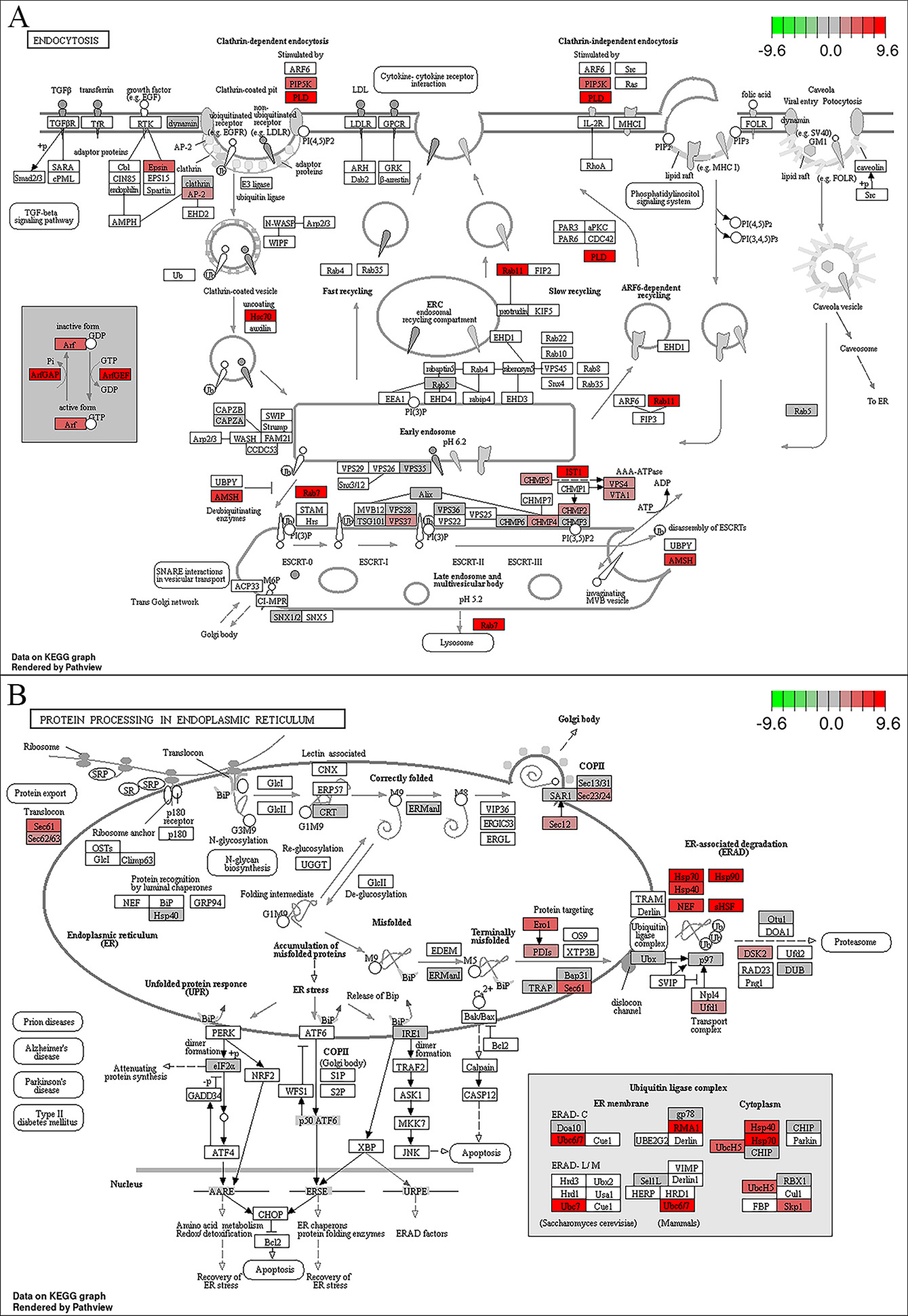


Figure S10. A: FPKM values of the core candidate DEGs. B: Average FPKM value of the core candidate DEGs. C: The FPKM ratio of up-regulated DEGs of M9T337 (T) or M.26 (M) dataset. The color intensity was proportional to the FPKM value. Taxa relative abundances were log10-transformed, and the scale method (from zero to one) was used for the heatmap representation. Each treatment included three repetitions.


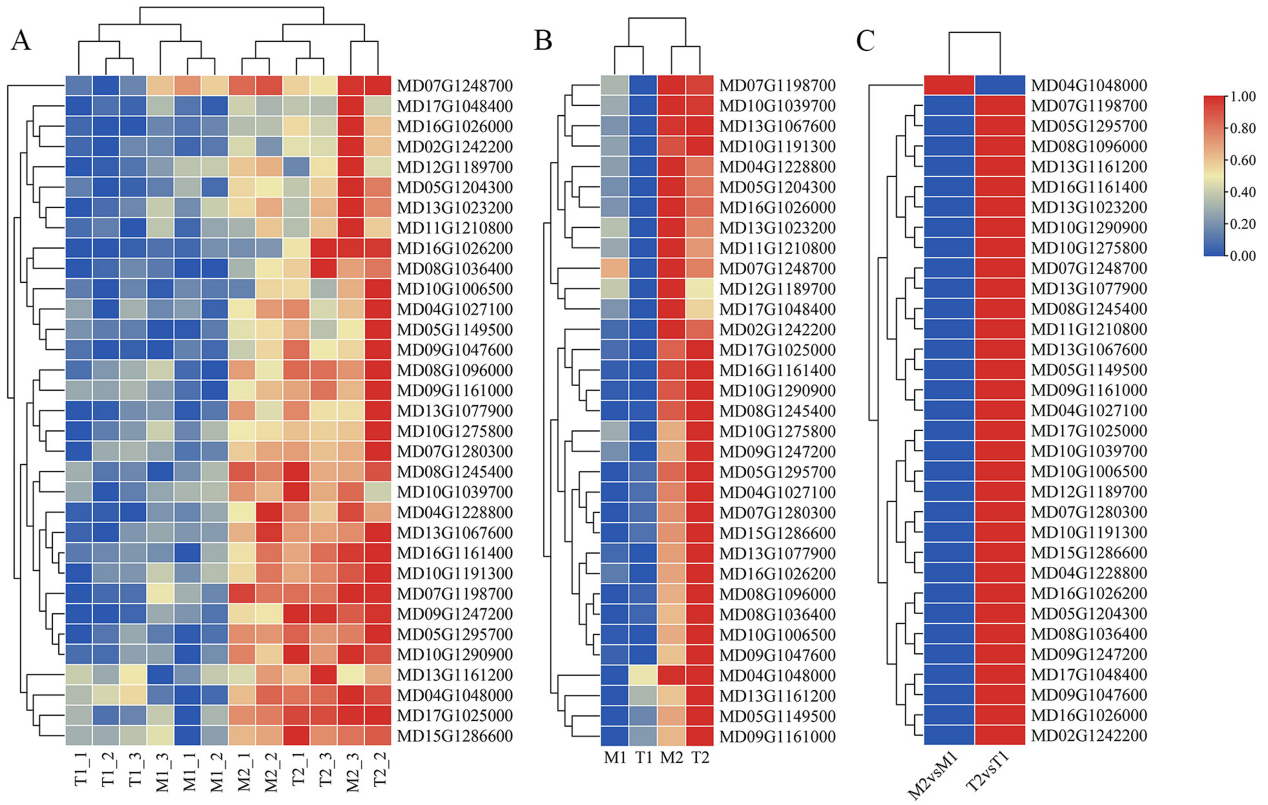

Supplement: Supplementary file 2 — Additional file 2: Supplementary Figures. [file 12864_2022_8721_MOESM2_ESM.docx]
